# Pulsed hyperfine EPR study of formally divalent rare earth (scandium, yttrium, lanthanum) arene complexes

**DOI:** 10.1039/d6cc06362d

**Published:** 2026-10-22

**Authors:** Tomáš Hajdu, Ross E. MacKenzie, Floriana Tuna, Conrad A. P. Goodwin, Eric J. L. McInnes

**Affiliations:** a Photon Science Institute, Centre for Radiochemical Research and Department of Chemistry, The University of Manchester Manchester M13 9PL UK eric.mcinnes@manchester.ac.uk

## Abstract

Pulsed EPR hyperfine spectroscopy of the formal rare earth(ii) arene complexes [RE^II^(NHAr^iPr6^)_2_] (2M, M = Sc, Y, La; NHAr^iPr6^ = {N(H)C_6_H_3_-2,6-(C_6_H_2_-2,4,6-^i^Pr_3_)_2_}), supported by DFT, reveals extensive delocalisation of spin density to the arene ring bound to the metal (56–73% Mulliken spin population, 42–53% Löwdin orbital population of SOMO, from DFT). Hence, they are RE(ii) complexes in a formal sense only.

## Abstract 2

Pulsed EPR of formal M^2+^ rare earth complexes, directly probing ligand spin density, shows they are better described as M^2.5+^.

## Graphical abstract

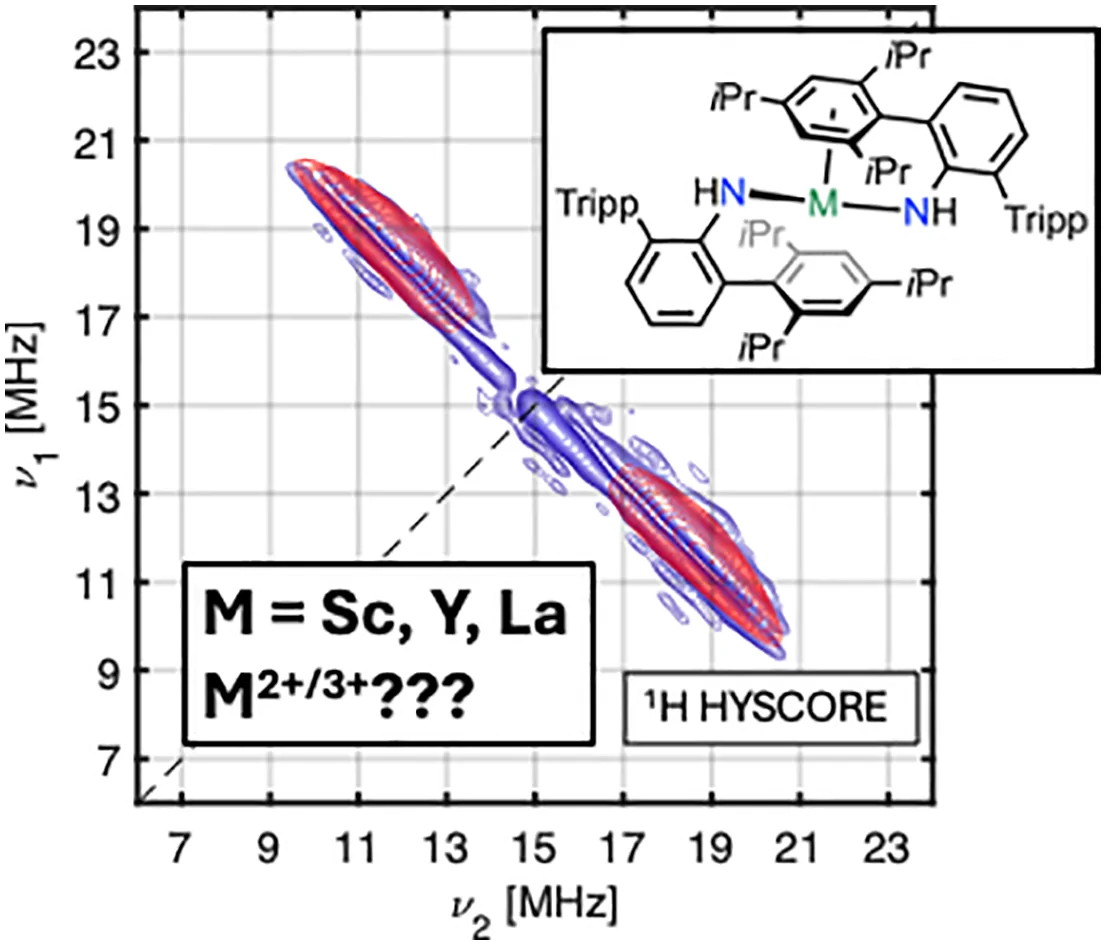

Rare earth (RE; Sc, Y, La–Lu) complexes in the uncommon RE(ii) oxidation state have attracted much interest.^1,2^ This is driven not just by the unusual electronic structures, but also by the consequent interesting magnetic properties^3^ and chemical reactivity.^4^ The majority of Sc(ii), Y(ii), and La(ii) examples isolated to date are trigonal complexes of general formula [RE^II^X_3_]^−^ where L = amide, aryloxide, and cyclopentadienide.^1^ There are fewer examples of RE(ii) in other geometries.^5,6^ The Sc, Y and La examples have a formal *n*d^1^ configuration (*n* = 3–5, respectively).^1,2^ In the trigonal crystal field of [RE^II^X_3_]^−^ the *n*d_*z*^2^_ orbital is stabilised, and extensive mixing with (*n* + 1)s can occur,^7^ and this leads to interesting electron spin relaxation properties.^8,9^

A new class of rare earth(2+) complex has been reported recently: [RE^II^(NHAr^iPr6^)_2_] [NHAr^iPr6^ = {N(H)C_6_H_3_-2,6-(C_6_H_2_-2,4,6-^i^Pr_3_)_2_}] which feature a highly asymmetric arene sandwich geometry supported by two bound amides (Fig. 1).^10–12^ Related complexes of O- and S-donor analogues of these ligands have been reported by Evans and co-workers,^13^ which build on works by Niemeyer.^14^ We, and others, have reported studies on the [RE^II^(NHAr^iPr6^)_2_] series where RE = Sc, Y, La (2RE).^10–12^ These have very different electronic structures to [RE^II^X_3_]^−^ type analogues. Continuous-wave (cw) EPR revealed weaker metal hyperfine interactions, indicating a significantly reduced degree of metal character to the singly occupied molecular orbital (SOMO).^10,11^ Moreover, this character was *n*d_*x*^2^−*y*^2^_ based, which could be interpreted as extensive metal-arene back donation.^11^ Here we use pulsed hyperfine EPR methods to directly probe this *via* the ligand hyperfine interactions, supported by *ab initio* calculations. There are few previous reports of pulsed EPR studies on RE(ii) complexes, and all have been on the [RE^II^X_3_]^−^ motiff:^8,9,15^ these have tended to focus on electron spin dynamics, although hyperfine data have been reported on [RE^II^(Cp′)_3_]^−^ (RE = Y, La, Lu; Cp′ = {C_5_H_4_(SiMe_3_)}), [Sc^II^{N(SiMe_3_)_2_}_3_]^−^, and [La^II^(Cp^R^)_3_]^−^ (Cp^R^ = Cp^tt^, Cp″ or Cp′; Cp^tt^ = {C_5_H_3_-1,3-^*t*^Bu_2_}, Cp″ = {C_5_H_3_-1,3-(SiMe_3_)_2_}).^8^

**Fig. 1:**
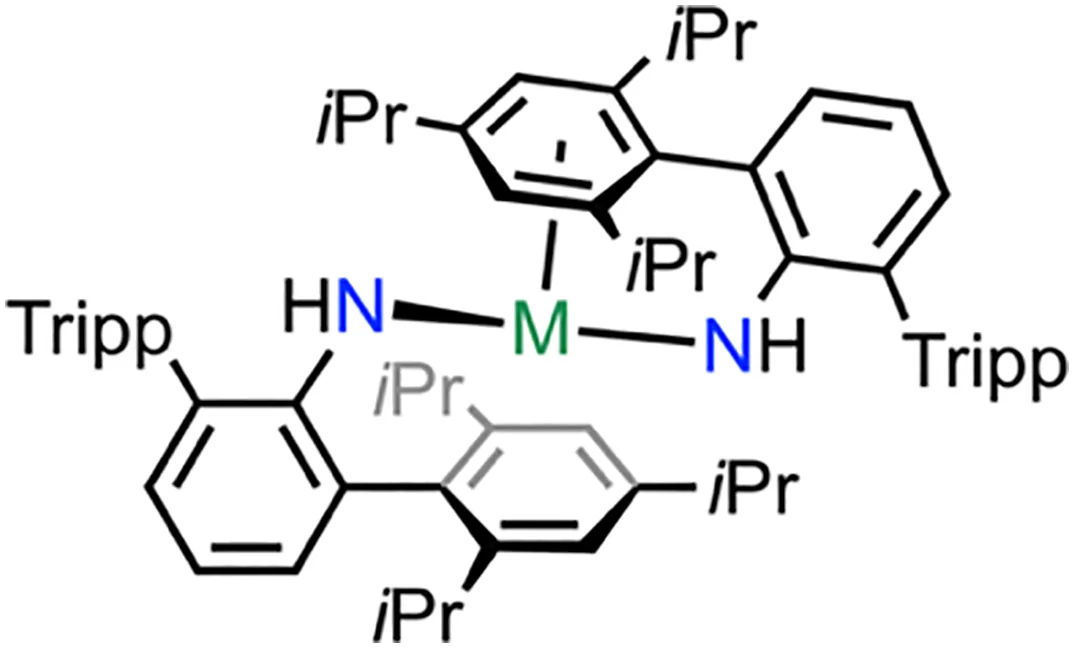
Schematic structure of 2RE (Tripp = −C_6_H_2_-2,4,6-^i^Pr_3_).

Complexes 2Sc and 2La have similar molecular structures from XRD,^11^ with the metal ion asymmetrically sandwiched between two Tripp {C_6_H_2_-2,4,6-^i^Pr_3_} arene groups, only binding strongly to one, and further bound to the amide arms of the central arene of each ligand (Fig. 1). The Tripp groups in 2Y are symmetrically bound in the solid state, but spectroscopic and computational studies show the structure relaxes in solution and more closely resembles the structures of 2Sc and 2La.^11^ The solid-state structures of 2Sc and 2La, and the calculated (solution) structure of 2Y, reveal that the closer-bound Tripp group has a fold of *ca.* 12° away from planarity.

Echo-detected field-swept (EDFS) X-band EPR spectra of 2Sc, 2Y and 2La in frozen ^*n*^Pr_2_O solution (Fig. 2) are consistent with cw EPR.^11^ The spectra are dominated by the metal hyperfine interaction (^45^Sc, *I* = 7/2; ^89^Y, *I* = 1/2; ^139^La, *I* = 7/2, all *ca.* 100% abundant), and axially symmetric within the resolution [2Sc: *g*_⊥_ = 2.002, *g*_‖_ = 1.990, *A*_⊥_ = 210, *A*_‖_ = 195 MHz; 2Y: *g*_⊥_ = 2.004, *g*_‖_ = 1.986, *A*_⊥_ = −39, *A*_‖_ = −36 MHz; 2La: *g*_⊥_ = 2.005, *g*_‖_ = 1.952, *A*_⊥_ = 110, *A*_‖_ = 100 MHz; note that although the relative signs of *A*_⊥_ and *A*_‖_ are fixed by their relationship to the experimental isotropic values, EPR does not give their absolute signs. The assignment here follows from the sign of the nuclear magnetic moments and as supported by DFT calculations].^11^ The *g*_⊥_ > *g*_‖_ pattern is consistent with a dominant d_*x*^2^−*y*^2^_ metal contribution to the SOMO, with the *g*-anisotropy increasing in the order 2Sc < 2Y < 2La. This trend follows the magnitude of the spin–orbit coupling (SOC) constant for the metal ions, although this is not linear because of significant differences in the metal character of the SOMO (see below).

**Fig. 2:**
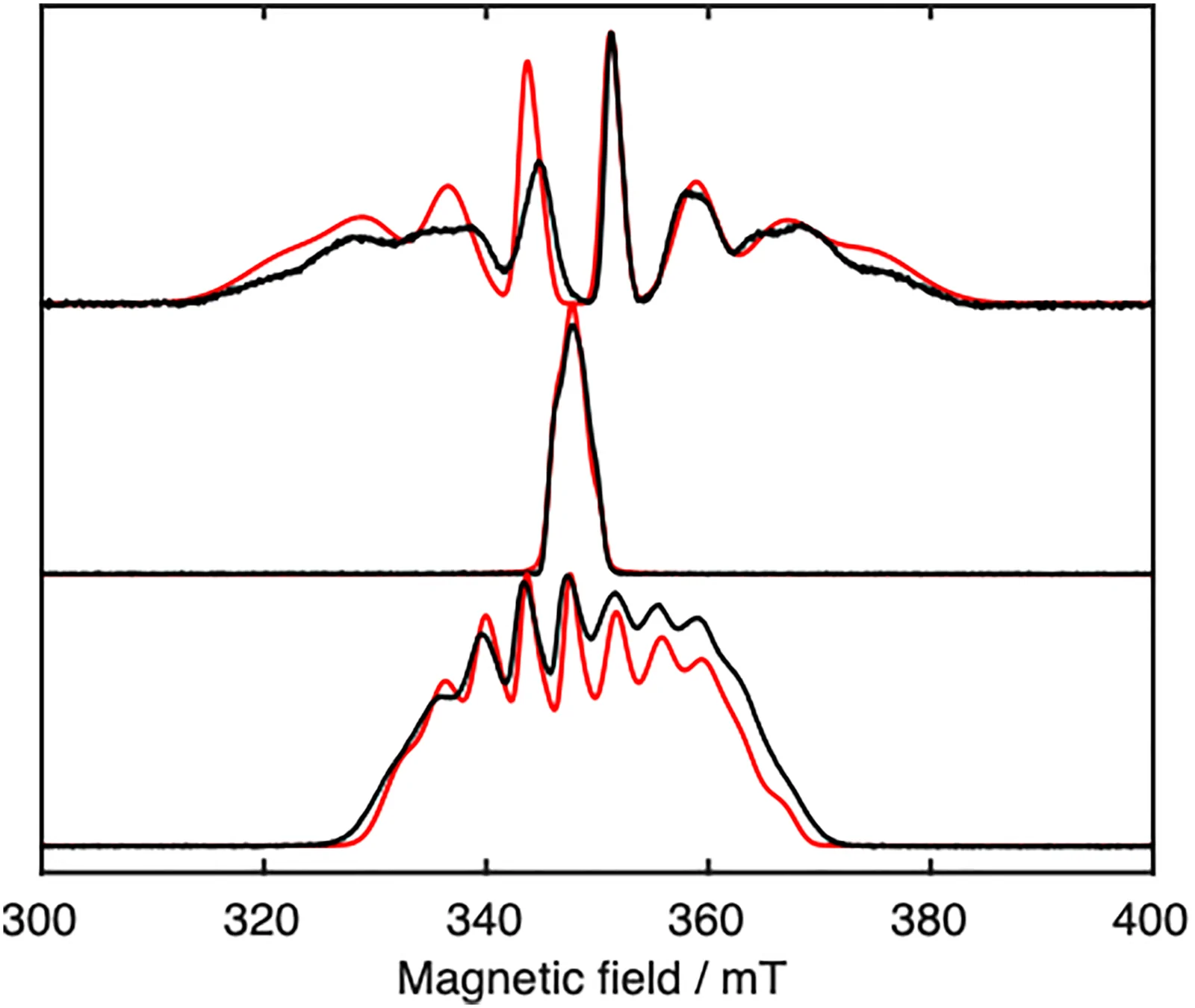
EDFS spectra of 2Sc (top; at 40 K), 2Y (middle; 20 K) and 2La (bottom; 20 K) in 1 mM ^*n*^Pr_2_O solution (black). Simulations (red) using parameters in the text.

As a precursor to pulsed hyperfine EPR experiments, we performed relaxation measurements. Inversion recovery experiments, measured at the maximum of the EDFS spectra at 20 K, give spin–lattice relaxation time constants of *ca.* 5.5, 1.1 and 0.8 ms for 2Sc, 2Y and 2La, respectively (Fig. S1–S7 and Table S1). This trend is consistent with increased SOC down the group, and with the increased *g*-anisotropy, as previously observed for the [RE^II^(Cp^R^)_3_]^−^ family.^8^ There is less dependence of the phase memory time constants on the identity of RE, being of the order of 1 µs from Hahn echo decay experiments (Fig. S1–S7).

X-band HYSCORE (hyperfine sublevel correlation)^16^ data for 2Sc, measured at the EDFS maximum, show signals from ^14^N and ^1^H nuclei in the (+, +) quadrant (Fig. S8) consistent with hyperfine interactions in the weakly coupled regime (*i.e.* 2*ν*_n_ > *A* where *ν*_n_ is the Larmor frequency at the applied magnetic field *B*_0_ and *A* is the hyperfine coupling). The ^1^H region (Fig. 3) comprises ridges with a maximum spread of *ca.* 10 MHz (*ν*_H_ ± 5.0 MHz; *ν*_H_ ≈ 15 MHz). A similar spread of frequencies is also observed in ENDOR (electron nuclear double resonance)^17^ spectra measured at the same *B*_0_ (Fig. S14).

**Fig. 3:**
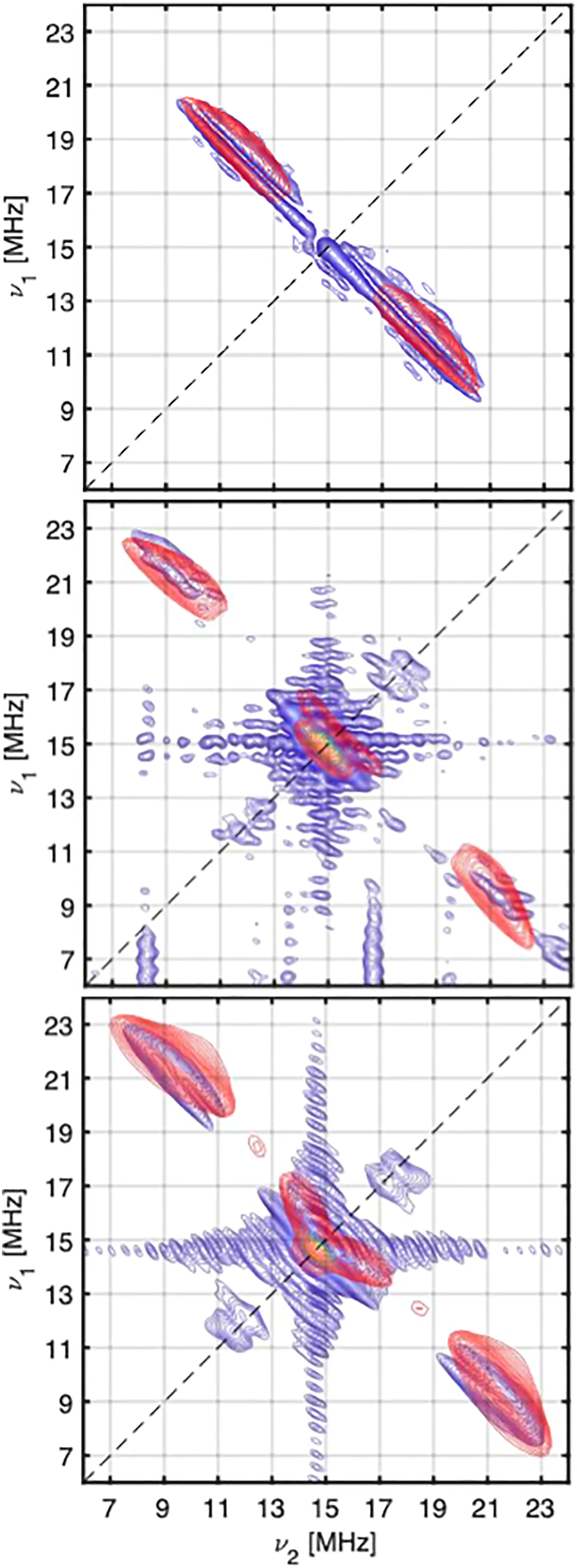
X-band HYSCORE spectra (^1^H region) of 2Sc (top; 40 K), 2Y (middle; 20 K) and 2La (bottom; 20 K) in 1 mM ^*n*^Pr_2_O solutions measured at the maximum of the EDFS spectra (blue). Calculated spectra (red) with hyperfine coupling to a single ^1^H nucleus (H2, Fig. 4) using parameters in the text and Table 2 and Tables S3–S5.

HYSCORE data for 2Y are more poorly resolved with lower signal-to-noise ratio, but ^14^N and ^1^H are again observed in the weakly coupled regime (Fig. 3 and Fig. S9). The islands due to ^1^H have a frequency spread of *ca.* 14 MHz (*ν*_n_ ± 7.0 MHz), *i.e.* wider than for 2Sc. These larger frequencies are also observed in ^1^H ENDOR (Fig. S15). HYSCORE (Fig. 3 and Fig. S10) and ENDOR (Fig. S16) data for 2La are similar to those for 2Y. In summary, the HYSCORE and ENDOR data reveal larger ligand ^1^H hyperfine interactions for 2Y and 2La than for 2Sc.

Because the spin distribution is nontrivial, we attempted to model the spectra using ligand hyperfine matrices calculated from DFT (see SI; calculations performed with ORCA^18^), based on the optimised structures.^11^

We previously reported calculations of the *g*- and metal hyperfine interactions,^11^ and we summarise the key points of the electronic structures here. We define the *z* axis as along the M⋯Tripp(centroid) direction for the bound Tripp group. The SOMO for all three complexes is a δ-bonding MO involving the metal valence *n*d_*x*^2^−*y*^2^_ and a π* orbital of the bound Tripp group (Fig. 4 and Fig. S17–S19). Löwdin population analysis gives the metal *n*d_*x*^2^−*y*^2^_ (*n*d_total_) contributions to the SOMO as 25% (34%) for 2Sc, 10% (19%) for 2Y, and 11% (14%) for 2La, respectively, consistent with the metal hyperfine interaction from cw EPR.^11^ Note that the metal character of the SOMO is significantly larger for 2Sc than for 2Y and 2La: this correlates with our initial observation that smaller ligand hyperfine interactions are observed for 2Sc than for 2Y and 2La. The calculated bound-Tripp π* character of the SOMO is 42% for 2Sc, 54% for 2Y, and 53% for 2La.

Mulliken spin population analysis for this bound arene ring shows a similar pattern in each case, with two C positions (C_C,F_ in Table 1 and Fig. 4) having small negative spin population, with the remaining four C positions (C_A,B,D,E_) carrying significant positive spin population. This corresponds to one of the 
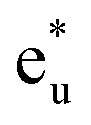
 π-orbitals of benzene (π_4_ in Fig. S20), which has the correct symmetry to overlap with the metal d_*x*^2^−*y*^2^_ orbital. For 2Sc the bulk of the ligand spin density is split between the C_A,B,D,E_ positions, while for 2Y and 2La the C_B,E_ positions dominate. The latter are the “hinge” of the fold of this arene (see above). Calculated spin populations for the N-atoms are negligible.

**Table 1:** Calculated Mulliken spin populations (%) for RE, and C-atoms of the bound arene in 2RE (see Fig. 4 for labelling)

	2Sc	2Y	2La
RE	45.3	30.9	29.9
C_A_	15.7	5.6	2.3
C_B_	14.6	31.3	35.4
C_C_	–2.3	–1.1	–1.4
C_D_	9.8	1.1	4.2
C_E_	22.0	38.7	33.4
C_F_	–4.2	–4.8	–0.7

**Fig. 4:**
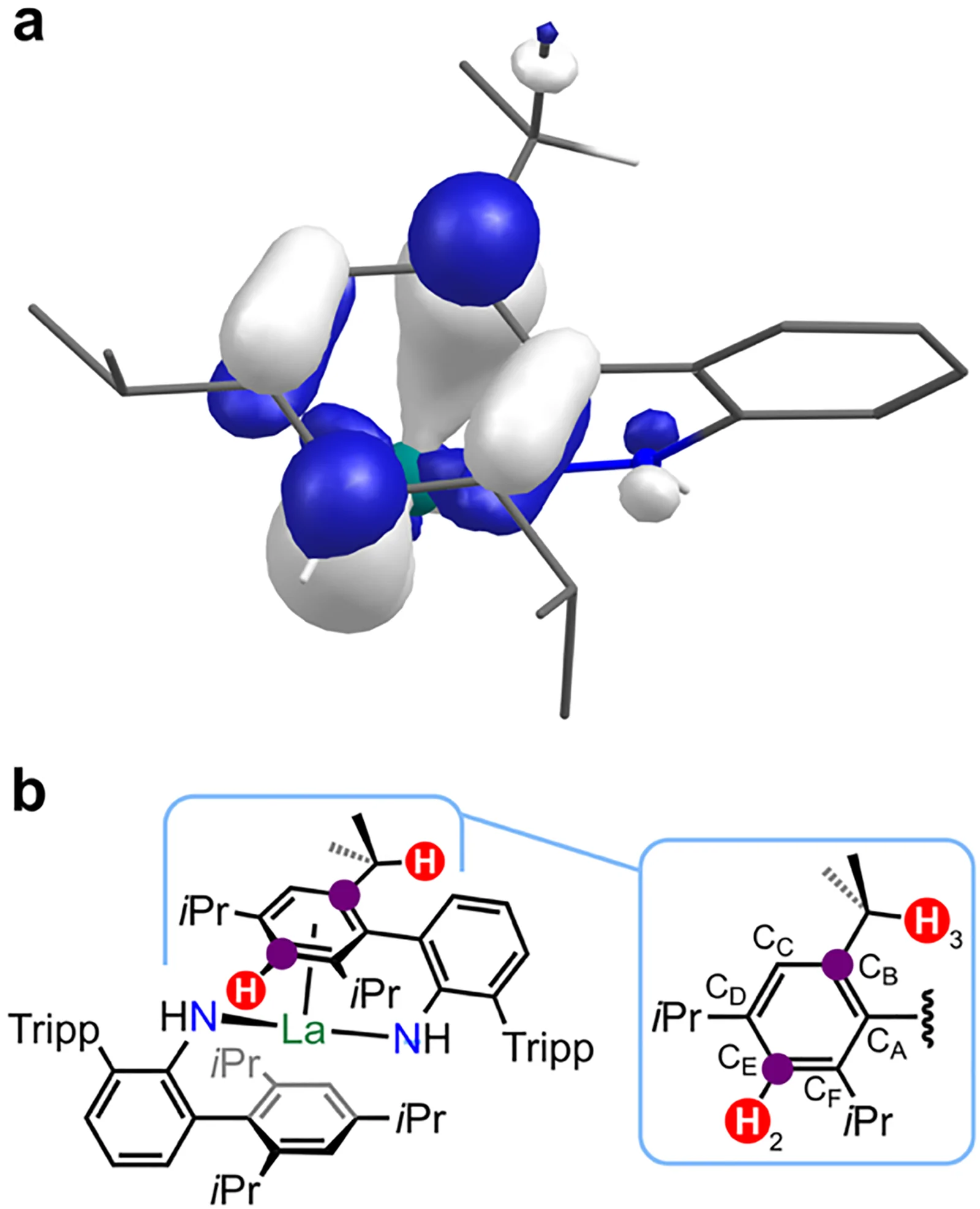
(a) Calculated SOMO of 2La (0.05 isosurface) viewed down the bound arene(centroid)-La vector (molecular *z* axis); for clarity only the bound arene and La are shown. (b) Schematic of structure highlighting the positions of the ^1^H nuclei that give the largest calculated hyperfine interactions (red circles), and Tripp ring hinge atoms (purple circles).

For all three complexes, the protons with the largest calculated isotropic hyperfine coupling constants (*A*_iso_) were identified: these are all associated with the bound arene (Fig. 4 and Fig. S17–S19). The principal values of the calculated *A*^H^ matrices are in Table 2, and their orientations with respect to the molecular *g*-tensor frame in Tables S3–S5.

**Table 2:** Calculated hyperfine matrices (principal values, listed in order of increasing magnitude; MHz) for the ^1^H associated with the C_B,E_ carbons (Fig. 4) of the bound arene in 2RE

		*A*_1_	*A*_2_	*A*_3_	*A*_iso_
2Sc	H2 (C_E_-H̲)	2.4	–3.0	–13.1	–4.6
	H3 (C_B_-CH̲Me_2_)	8.8	9.4	15.4	11.2
2Y	H2 (C_E_-H̲)	2.5	–4.0	–17.0	–6.2
	H3 (C_B_-CH̲Me_2_)	1.5	2.2	8.3	4.0
2La	H2 (C_E_-H̲)	0.8	–5.9	–19.7	–8.2
	H3 (C_B_-CH̲Me_2_)	3.1	3.8	10.1	5.7

For 2Y and 2La the largest ^1^H hyperfine is found for the α-^1^H bonded to C_E_ (H2) which carries significant spin density (Table 1). The largest magnitude principal value of the hyperfine matrix for the H2 position is −13, −17 and −20 MHz for 2Sc-2La, respectively (Table 2). This is consistent with the narrowest ^1^H hyperfine ridges observed in HYSCORE being for 2Sc (see above). In each of 2RE, the orientation of this hyperfine component is near parallel to the cross product of the C–H and C 2p_π_ vectors (Fig. S21–S23). This is expected for α-protons in organic π-radicals where the isotropic part of the hyperfine interaction arises from spin polarisation of the C–H bond.^19^ The next largest hyperfine values belong to the methine ^1^H of the ^i^Pr group bound to C_B_ (H3), which carries similar spin density to C_E_ (Tables 1 and 2). It is a well-known feature of organic π-radicals that β-^1^H can give similar isotropic coupling to α-^1^H for a similar C spin density.^20^ In each of 2Sc-2La, the only other α-^1^H (H5) gives small hyperfine couplings (Table S2) due to the small spin density at the associated carbon (C_C_).

Calculated ^1^H HYSCORE spectra^21^ including only H2 account for the major ridges (outside the |*A*| ≈ *ν*_H_ region where there are contributions from many weakly coupled ^1^H) in the experimental spectra (Fig. 3; H3 gives peaks at the outer limits of the ridges for 2Sc, see Fig. S11). Including all of H1–H4 gives slightly better agreement with the HYSCORE (Fig. S11–S13). Calculated ENDOR spectra for the individual ^1^H sites, and combined, show that H2 and H3 dominate beyond the *ν*_H_ ± 3 MHz regions (Fig. S14–S16; we find better agreement to ENDOR peak positions and relative intensities allowing the hyperfine to H3 to vary by up to 20% from the DFT values, this makes little difference to calculated HYSCORE). There is little sensitivity to the orientations because there is limited orientation selectivity in the spectra. Overall, the calculated hyperfine data, and hence the calculated spin densities, agree well with the experimental hyperfine spectroscopy.

In conclusion, we have reported a pulsed EPR hyperfine study on low symmetry, [RE^II^(NHAr^iPr6^)_2_] (2RE, M = Sc, Y, La) complexes with formal RE(ii) oxidation states and *n*d^1^ electron configurations. cw EPR data confirm the dominant *n*d_*x*^2^−*y*^2^_ metal contribution to the SOMO. Pulsed hyperfine EPR methods give direct confirmation of extensive spin density on the bound arene. The computational results, validated by agreement of calculated ligand hyperfine data with experimental spectra, show that over half the spin density is associated with the bound arene. Despite this, the cw EPR spectra are dominated by the metal hyperfine interaction. In terms of orbital populations, the SOMO is just under half arene in character for 2Sc, and just over half for 2Y and 2La, due to extensive *n*d_*x*^2^−*y*^2^_ – π*(Tripp) mixing *via* a δ-bonding interaction. Hence, the formal RE(ii) complexes 2RE are equally validly (or invalidly) formulated as [RE^III^(NHAr^iPr6^)^−^(NHAr^iPr6^)^2−^], where the closer arene ligand has been reduced to an anion radical state. Neither crude description is satisfactory. Highly reduced rare earth complexes featuring metal–arene contacts are a burgeoning area of research,^22^ and the results herein provide a multi-pronged approach towards understanding the nature of the metal ion in these species.

## Conflicts of interest

There are no conflicts to declare.

## Supplementary Material

CC-OLF-D6CC06362D-s001

## Data Availability

Raw instrument and calculation outputs are deposited at FigShare (DOI: https://doi.org/10.48420/33965086). All other data are available in the supplementary information (SI). Supplementary information is available. See DOI: https://doi.org/10.1039/d6cc06362d.

## References

[cit1] Wedal J. C., Evans W. J. (2021). J. Am. Chem. Soc..

[cit2] Hitchcock P. B., Lappert M. F., Maron L., Protchenko A. V. (2008). Angew. Chem., Int. Ed..

[cit3] Meihaus K. R., Fieser M. E., Corbey J. F., Evans W. J., Long J. R. (2015). J. Am. Chem. Soc..

[cit4] Evans W. J., Lee D. S., Lie C., Ziller J. W. (2004). Angew. Chem., Int. Ed..

[cit5] Gould C. A., McClain K. R., Yu J. M., Groshens T. J., Furche F., Harvey B. G., Long J. R. (2019). J. Am. Chem. Soc..

[cit6] Griffin L. P. , Cameron I. J., Seed J. A., Whitehead G. F. S., McInnes E. J. L. and Goodwin C. A. P., *ChemRxiv*, 2026 https://dx.doi.org/10.26434/chemrxiv.15001204/v1

[cit7] MacDonald M. R., Ziller J. W., Evans W. J. (2011). J. Am. Chem. Soc..

[cit8] Ariciu A.-M., Woen D. H., Huh D. N., Nodaraki L. E., Kostopoulos A. K., Goodwin C. A. P., Chilton N. F., McInnes E. J. L., Winpenny R. E. P., Evans W. J., Tuna F. (2019). Nat. Commun..

[cit9] Kundu K., White J. R. K., Moehring S. A., Yu J. M., Ziller J. W., Furche F., Evans W. J., Hill S. (2022). Nat. Chem..

[cit10] Jena R., Benner F., Delano IV F., Holmes D., McCracken J., Demir S., Odom A. L. (2023). Chem. Sci..

[cit11] MacKenzie R. E., Hajdu T., Seed J. A., Whitehead G. F. S., Adams R. W., Chilton N. F., Collison D., McInnes E. J. L., Goodwin C. A. P. (2024). Chem. Sci..

[cit12] Benner F., Jena R., Odom A. L., Demir S. (2025). J. Am. Chem. Soc..

[cit13] Gilbert-Bass K., Stennett C. R., Grotjahn R., Ziller J. W., Furche F., Evans W. J. (2024). Chem. Commun..

[cit14] Niemeyer M. (2001). Eur. J. Inorg. Chem..

[cit15] Smith P. W., Hrubý J., Evans W. J., Hill S., Minasian S. G. (2024). J. Am. Chem. Soc..

[cit16] van Doorslaer S. (2017). eMagRes.

[cit17] Harmer J. (2016). eMagRes.

[cit18] Neese F. (2012). Wiley Interdiscip. Rev.: Comput. Mol. Sci..

[cit19] Atherton N. M. , Principles of electron spin resonance, Ellis Horwood, 1993

[cit20] Gerson F. and Huber W., Electron spin resonance spectroscopy of organic radicals, Wiley-VCH, 2003

[cit21] Stoll S., Schweiger A. (2006). J. Magn. Reson..

[cit22] Wang Y., Liang J., Deng C., Sun R., Fu P.-X., Wang B.-W., Gao S., Huang W. (2023). J. Am. Chem. Soc..

