# Supplementary material for: Pulsed hyperfine EPR study of formally divalent rare earth (scandium, yttrium, lanthanum) arene complexes
Source: Chem Commun (Camb). 2026 Oct 22. Online ahead of print. doi: 10.1039/d6cc06362d (PMC13711156; doi:10.1039/d6cc06362d)
Supplement: Supplementary file 1 [file CC-OLF-D6CC06362D-s001.pdf]

Supplementary Information (SI) for Chemical Communications.
This journal is © The Royal Society of Chemistry

Supplementary Information: Pulsed hyperfine EPR study of formally divalent rare earth (scandium, yttrium, lanthanum) arene complexes

Tomáš Hajdu, Ross E. MacKenzie, Floriana Tuna, Conrad A. P. Goodwin and Eric J. L. McInnes

Methods

Materials were prepared as reported in ref [1].

EPR spectroscopy

Samples for EPR measurements were prepared as frozen 1 mM nPr$_2$O solutions of [RE^{II}(NHAr^{iPr6})$_2$] complexes (RE = Sc, Y, and La), which were flash frozen in liquid nitrogen under inert atmosphere and then flame sealed in quartz tubes under vacuum. The pulsed EPR spectra were recorded as frozen solutions with a Bruker ElexSys E580 spectrometer operating at X–band frequency (ca. 9.7 GHz) equipped with a Cryogenic closed-system helium cryostat. For ENDOR experiments a radiofrequency (RF) bridge E 560-P Pulsed ENDOR Accessory was used. For **2Y** and **2La**, all pulsed experiments were performed at 20 K. For **2Sc**, experiments at 20 K were hampered by long spin-lattice relaxation time constants (see Figure S1) which limited the possible shot repetition time; therefore, hyperfine measurements were performed at 40 K.

Echo-detected field-swept (EDFS) spectra were measured with a (π/2–τ–π–τ–echo) echo with 16 ns π/2 pulses and τ = 300 ns. Phase memory time constant (T_m) measurements used a primary Hahn–echo sequence (π/2–τ–π–τ–echo) with varying inter–pulse delay τ. Data were fitted to:

$$Y(2\tau) = Y(0)e^{(-2\tau/T_m)^x}$$

where $Y(2\tau)$ is the echo intensity for delay τ, $Y(0)$ is the intensity extrapolated to τ = 0, and x is a stretch parameter. Spin–lattice relaxation time constant (T_1) measurements used the inversion recovery sequence (π–t–π/2–τ–π–τ–echo) with 16 and 32 ns π/2 and π pulse lengths, respectively, and τ = 200 ns. Data were fitted to:

$$Y(t) = Y_1 e^{(-t/T_1)}$$

where Y_1 is the amplitude.

HYSCORE measurements used a four–pulse ESEEM sequence (π/2–τ–π/2–t_1–π–t_2–π/2–echo) with 16 ns π/2 pulses, τ = 130 (**2Sc**) or 170 ns (**2Y**, **2La**), initial $t_{1,2}$ = 0.110 μs and increment 8 ns with 256 data points in both dimensions. A four-step phase–cycle was used. HYSCORE is a 2D ESEEM technique that correlates nuclear frequencies in the α and β electron spin manifolds.[2] In the (+,+) region of the 2D domain weakly coupled nuclei ($2|\upsilon_n| > |A|$) peaks appear centred at the Larmor frequency υ_n of the nucleus and separated by the hyperfine coupling constant A. ENDOR spectroscopy used the Mims sequence (π/2–τ–π/2–π_{RF}–π/2–echo) with π/2 pulse lengths of 16 ns and τ = 160 ns. The Mims ENDOR sequence introduces blind spots in the spectra at $A = n/\tau$ (n = integer; A in frequency units):[3] these are marked by arrows in Figures S14, S16, S18. Other τ values were measured to test the model.
All calculated EPR spectra were performed with EasySpin software.[4]

Computational

We previously reported *ab initio* calculations of the g- and metal hyperfine matrices for **2RE**.[1] Scalar relativistic unrestricted Kohn-Sham DFT calculations were performed. Partial geometry optimisations were performed on **2Sc** and **2La** using the TPSSh hybrid meta-generalised gradient density functional (meta-GGA)[5,6] with Grimme's D3BJ dispersion correction,[7-9] and the resolution of the identity 'chain of spheres' (RIJCOSX) approximation.[10,11] In the case of **2Y**, a full geometry optimisation was performed using an Et_2O solvent continuum model (CPCM) starting with **2La** as the starting structure. The resultant geometry was found to be a true local minimum by harmonic frequency calculations and deviated from the structure derived from SC-XRD analysis by having a single arene ring distorted (like **2La**) rather than two as in the SC-XRD structure. Calculations with this structure were found to better reproduce the solution spectroscopic features of **2Y** than the solid state structure (see previous work for more details).[1] Segmented all-electron relativistically contracted (SARC) basis sets were used for Sc (ma-ZORA-def2-QZVPP), Y (SARC-ZORA-TZVPP), and La (SARC2-ZORA-QZVP),[12-14] and appropriate relativistically re-contracted basis sets were used with H, C, N (ZORA-def2-TZVP). A SARC/J auxiliary basis set was used for each atom type as appropriate.[15,16] No symmetry constraints were imposed. Occasionally the SlowConv switch was used to ensure SCF convergence, along with the DefGrid3 (tight integration grid) switch. In the case of EPR calculations, alternative basis sets for the metal ions were explored without improvement in the results. All were used with their default settings within ORCA 6.

Table S1. Spin lattice relaxation (T_1) and phase memory relaxation (T_m) time constants of **2Sc**, **2Y**, and **La**.

	**2Sc** (40 K)	**2Sc** (20 K)	**2Y** (20 K)		**2La** (20 K)		
B_0 /mT	**350.0**	**350.0**	**341.5**	**343.0**	**342.0**	**349.3**	**368.0**
T_1/ms	0.537(2)[b]	5.5(1)[a]	1.516(1)	1.145(1)	0.611(1)	0.323(2)	0.813(4)
T_m/μs	0.546(8)[b]		1.274(4)	1.171(3)	1.200(4)	1.259(3)	1.146(10)
x	0.77(1)		1.27(1)	1.11(1)	1.15(1)	1.25(1)	1.08(1)

(a) estimated from data in Fig. S1; further pulsed data for **2Sc** were measured at higher temperatures due to the long T_1. (b) uncertainties from statistical fitting.

Table S2. Principal values of the largest calculated ^{1}H hyperfine (**A**, MHz) coupling matrices: see Tables S3-S5 for labelling.

**Complex**	**C position**	**Atom**	A_1	A_2	A_3	A_{iso}
**2Sc**	C_D (methine)	H1	2.65	2.78	4.38	3.27
	C_E (α-^{1}H)	H2	2.41	–3.03	–13.13	–4.59
	C_B (methine)	H3	8.77	9.36	15.36	11.16
	C_B (methyl)	H4	2.26	3.00	5.26	3.50
	C_C (α-^{1}H)	H5	–0.88	2.40	–2.89	–0.45
**2Y**	C_D (methine)	H1	3.10	3.20	4.84	3.71
	C_E (α-^{1}H)	H2	2.46	–3.98	–16.96	–6.16
	C_B (methine)	H3	1.53	2.19	8.34	4.02
	C_B (methyl)	H4	3.77	4.72	7.18	5.22
	C_C (α-^{1}H)	H5	1.06	–3.28	–5.07	–2.43
**2La**	C_D (methine)	H1	2.34	2.35	6.84	3.85
	C_E (α-^{1}H)	H2	0.84	–5.86	–19.68	–8.23
	C_B (methine)	H3	3.12	3.77	10.09	5.66
	C_B (methyl)	H4	4.47	5.42	7.99	5.96
	C_C (α-^{1}H)	H5	1.08	–2.56	–4.18	–1.89

Table S3. Direction cosine matrices relating calculated ^{1}H hyperfine matrix (***A***; as listed in Table 2) and ***g***-matrix principal axes for **2Sc** (molecular coordinates are in ref. [1]). Right: schematic of ^{1}H labels.

	A_1g_1	A_1g_2	A_1g_3	A_2g_1	A_2g_2	A_2g_3	A_3g_1	A_3g_2	A_3g_3
**H2**	–0.8871	0.3180	–0.3347	0.3122	–0.1209	0.9423	–0.3401	–0.9404	–0.0079
**H3**	–0.6522	–0.7577	–0.0241	–0.0006	–0.0312	0.9995	–0.7580	0.6519	0.0199
**H4**	–0.3348	–0.9141	–0.2286	–0.3818	–0.0902	0.9198	–0.8615	0.3953	–0.3189
**H5**	–0.7360	0.5279	0.4238	–0.6729	–0.6391	–0.3725	0.0742	–0.5594	–0.8256
**H1**	–0.8580	–0.2779	0.4320	0.5125	–0.4076	0.7557	–0.0339	0.8698	0.4922

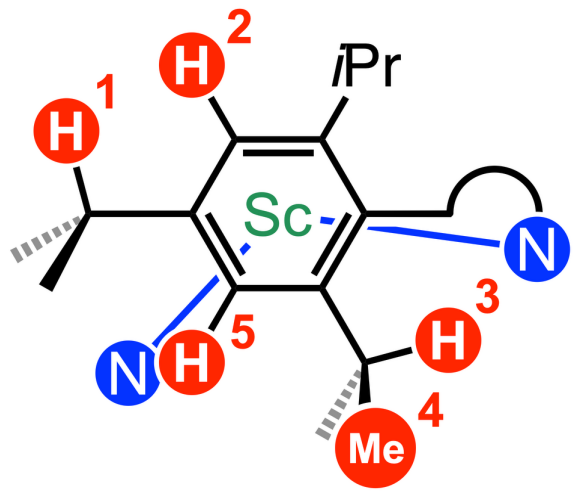

Table S4. Direction cosine matrices relating calculated ^{1}H hyperfine matrix (***A***; as listed in Table 2) and ***g***-matrix principal axes for **2Y** (molecular coordinates are in ref. [1]). Right: schematic of ^{1}H labels.

	A_1g_1	A_1g_2	A_1g_3	A_2g_1	A_2g_2	A_2g_3	A_3g_1	A_3g_2	A_3g_3
**H2**	0.9595	–0.2414	0.1454	–0.1379	0.0476	0.9893	–0.2457	–0.9693	0.0124
**H3**	–0.6020	–0.7848	–0.1471	–0.3957	0.1332	0.9087	0.6935	–0.6052	0.3907
**H4**	–0.3502	–0.9267	0.1361	–0.5899	0.3311	0.7365	0.7276	–0.1776	0.6626
**H5**	–0.5663	–0.7302	–0.3823	0.8214	–0.4620	–0.3345	0.0677	–0.5034	0.8614
**H1**	0.9582	0.2793	–0.0620	0.1396	–0.2672	0.9535	0.2498	–0.9223	–0.2950

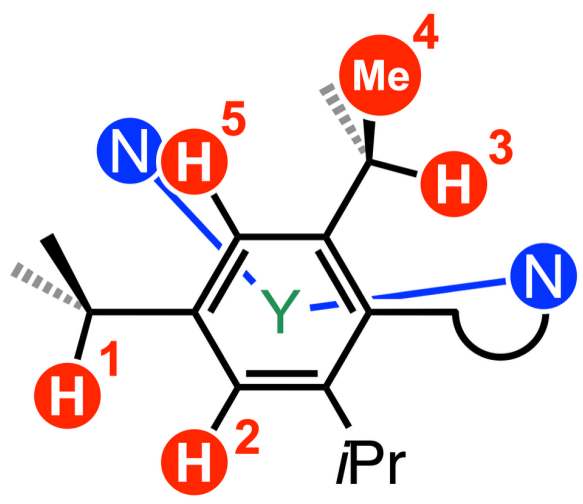

Table S5. Direction cosine matrices relating calculated ^{1}H hyperfine matrix (***A***; as listed in Table 2) and ***g***-matrix principal axes for **2La** (molecular coordinates are in ref. [1]). Right: schematic of ^{1}H labels.

	A_1g_1	A_1g_2	A_1g_3	A_2g_1	A_2g_2	A_2g_3	A_3g_1	A_3g_2	A_3g_3
**H2**	–0.9774	0.1798	–0.1116	–0.1199	–0.0357	0.9922	0.1744	0.9831	0.0564
**H3**	0.5368	0.8121	0.2289	–0.3632	–0.0224	0.9314	–0.2828	0.5831	–0.2828
**H4**	0.2704	0.9613	–0.0524	–0.5790	0.2058	0.7889	–0.7692	0.1830	–0.6122
**H5**	0.4811	0.7676	0.4235	–0.8759	0.4008	0.2686	0.0364	–0.5001	0.8652
**H1**	–0.4886	–0.3112	0.8152	–0.7457	–0.3362	–0.5753	–0.4531	0.8889	0.0677

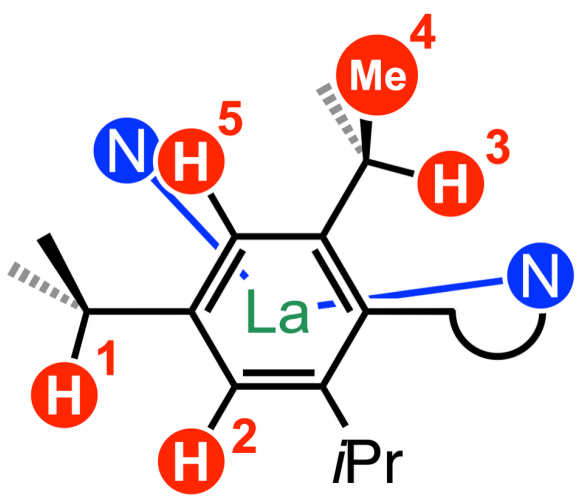

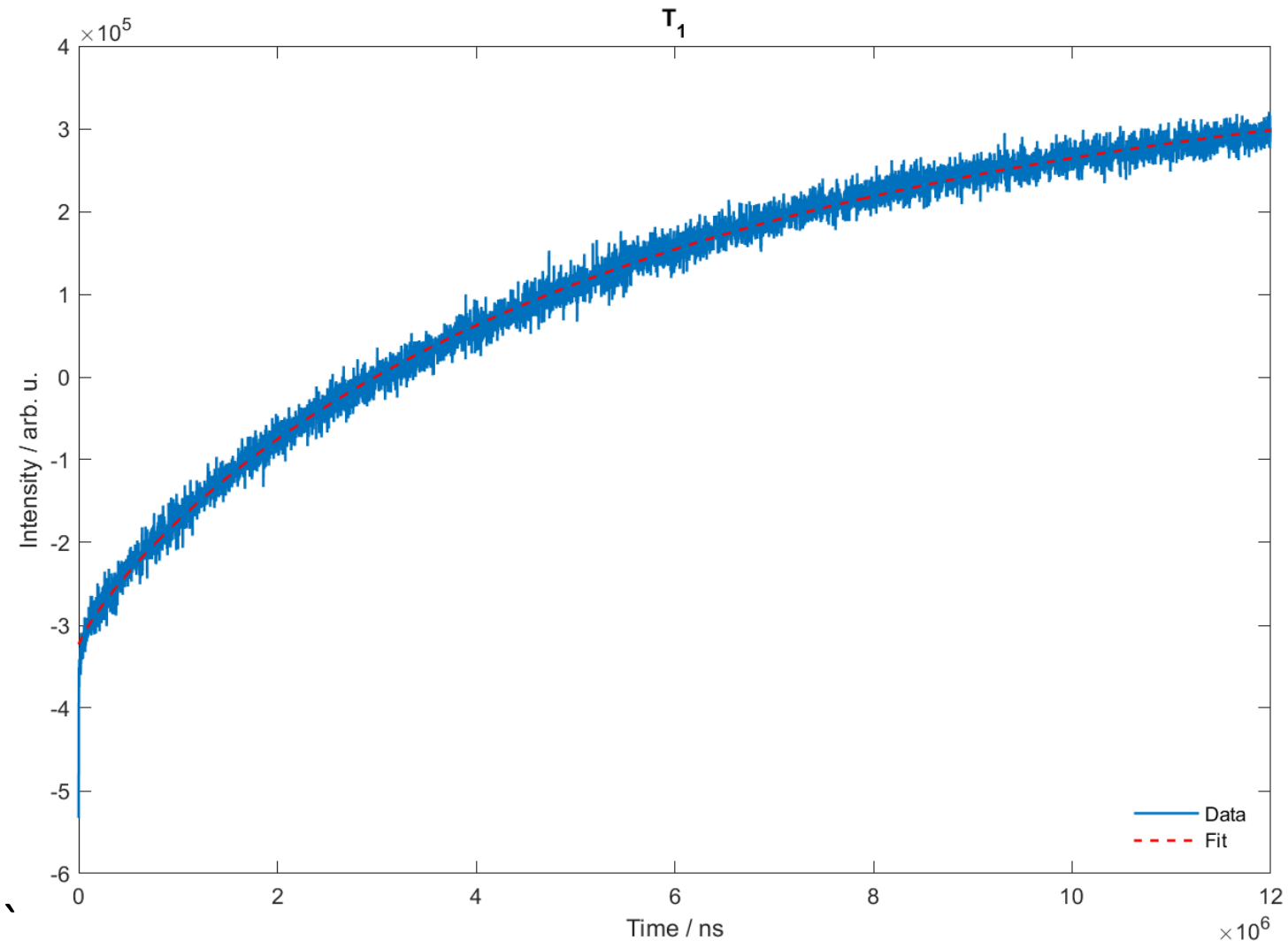

Figure S1. Spin-lattice relaxation time (T_1) data for a 1 mM ^{n}Pr$_2$O frozen solution of **2Sc** measured at B_0 = 350.0 mT and at 20 K.

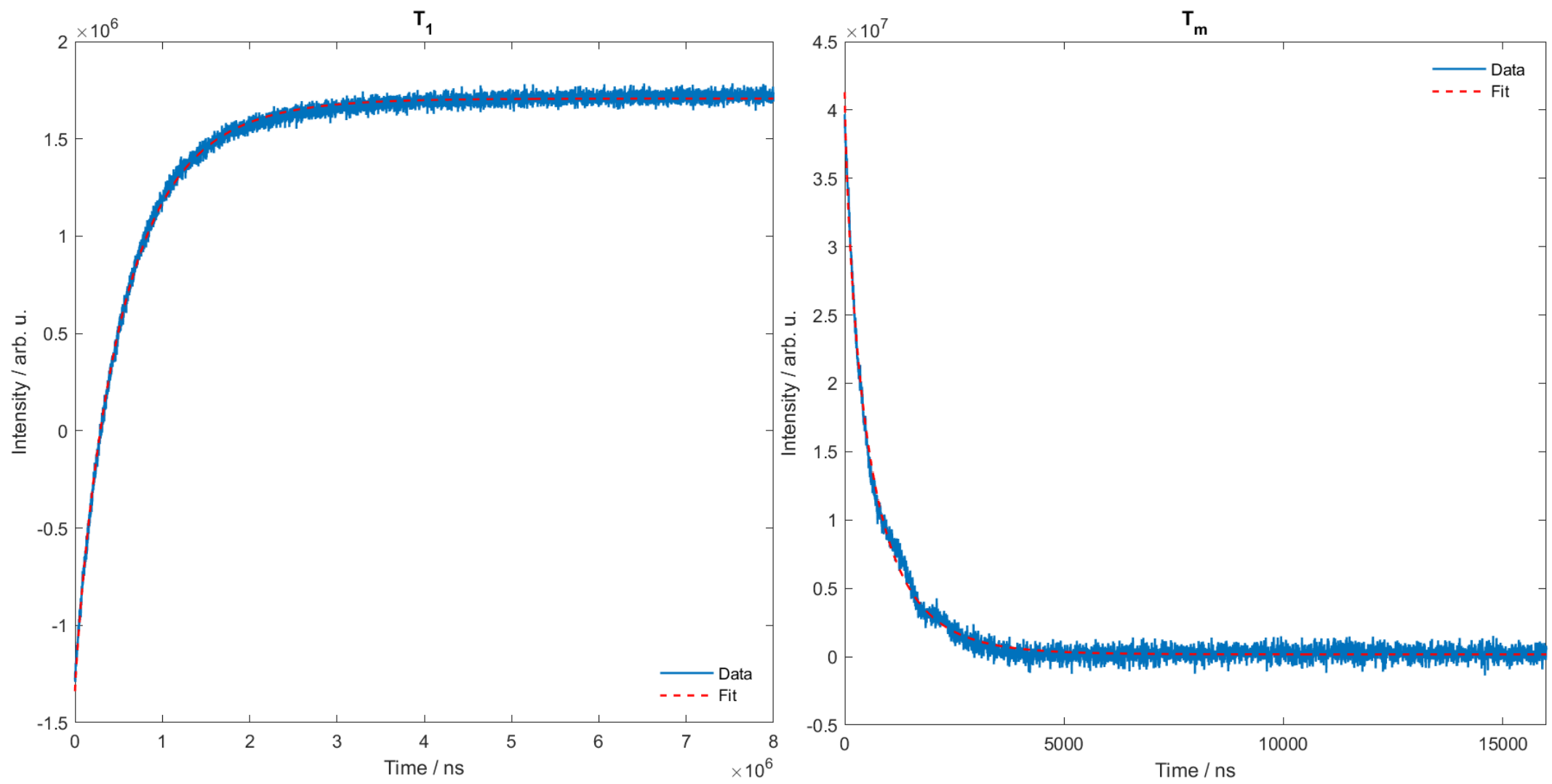

Figure S2. Spin lattice (T_1; left) and phase memory (T_m; right) relaxation time data for a 1 mM ^{n}Pr$_2$O frozen solution of **2Sc** measured at B_0 = 350.0 mT and at 40 K.

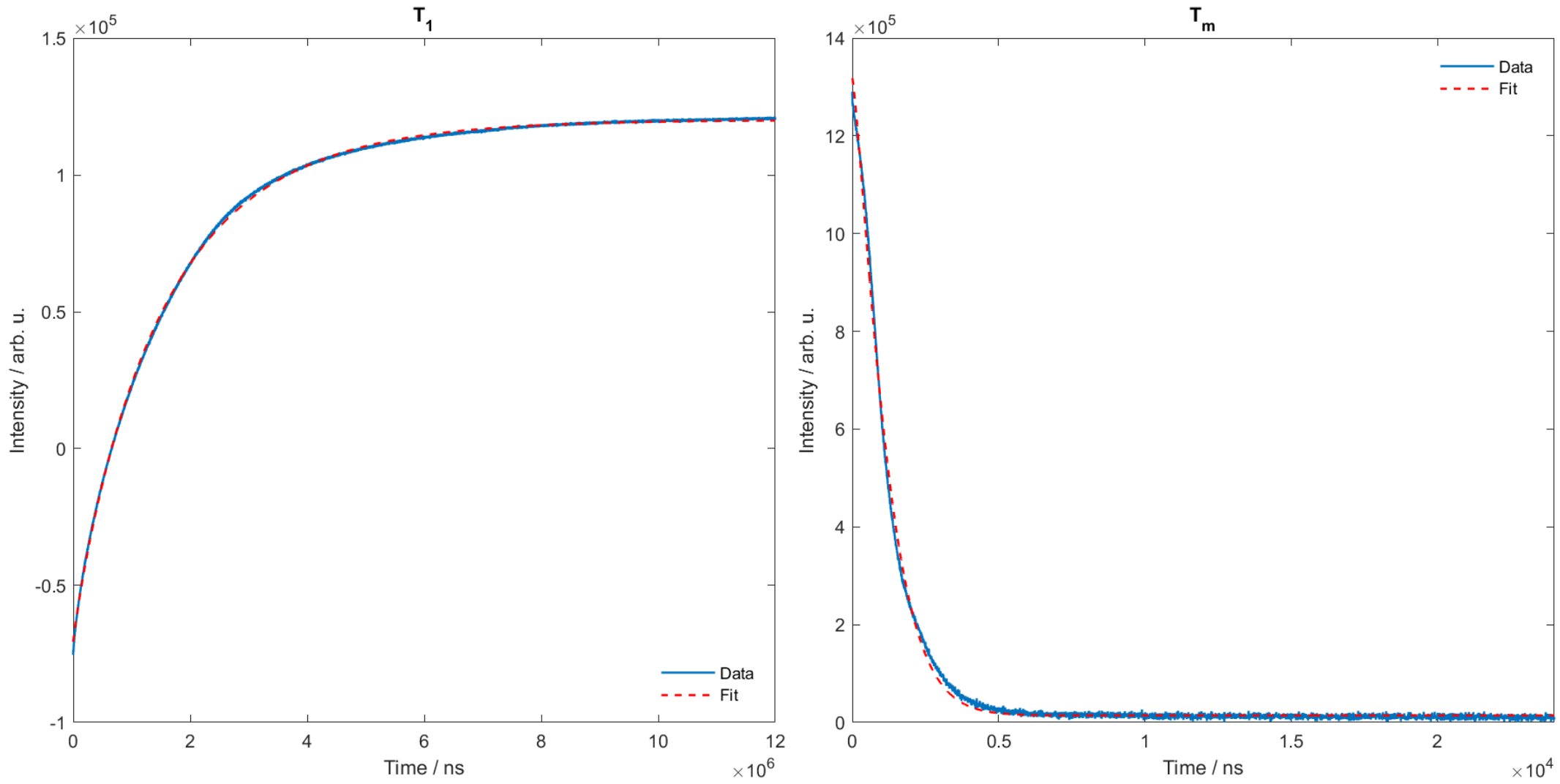

Figure S3. Spin lattice (T_1; left) and phase memory (T_m; right) relaxation time data for a 1 mM $^{n}Pr_2O$ frozen solution of **2Y** measured at B_0 = 341.5 mT and at 20 K.

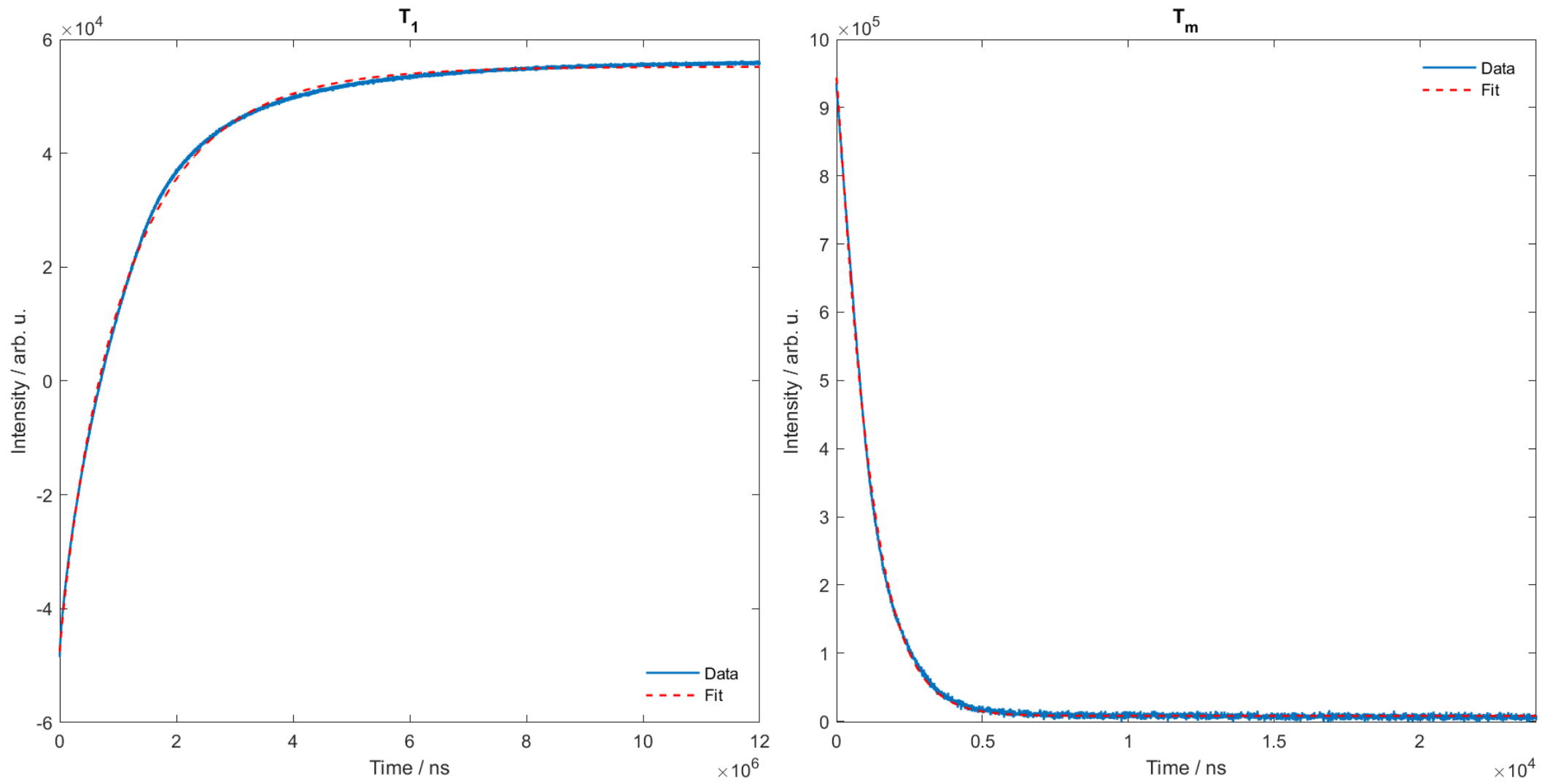

Figure S4. Spin lattice (T_1; left) and phase memory (T_m; right) relaxation time data for a 1 mM $^{n}Pr_2O$ frozen solution of **2Y** measured at B_0 = 343.0 mT and at 20 K.

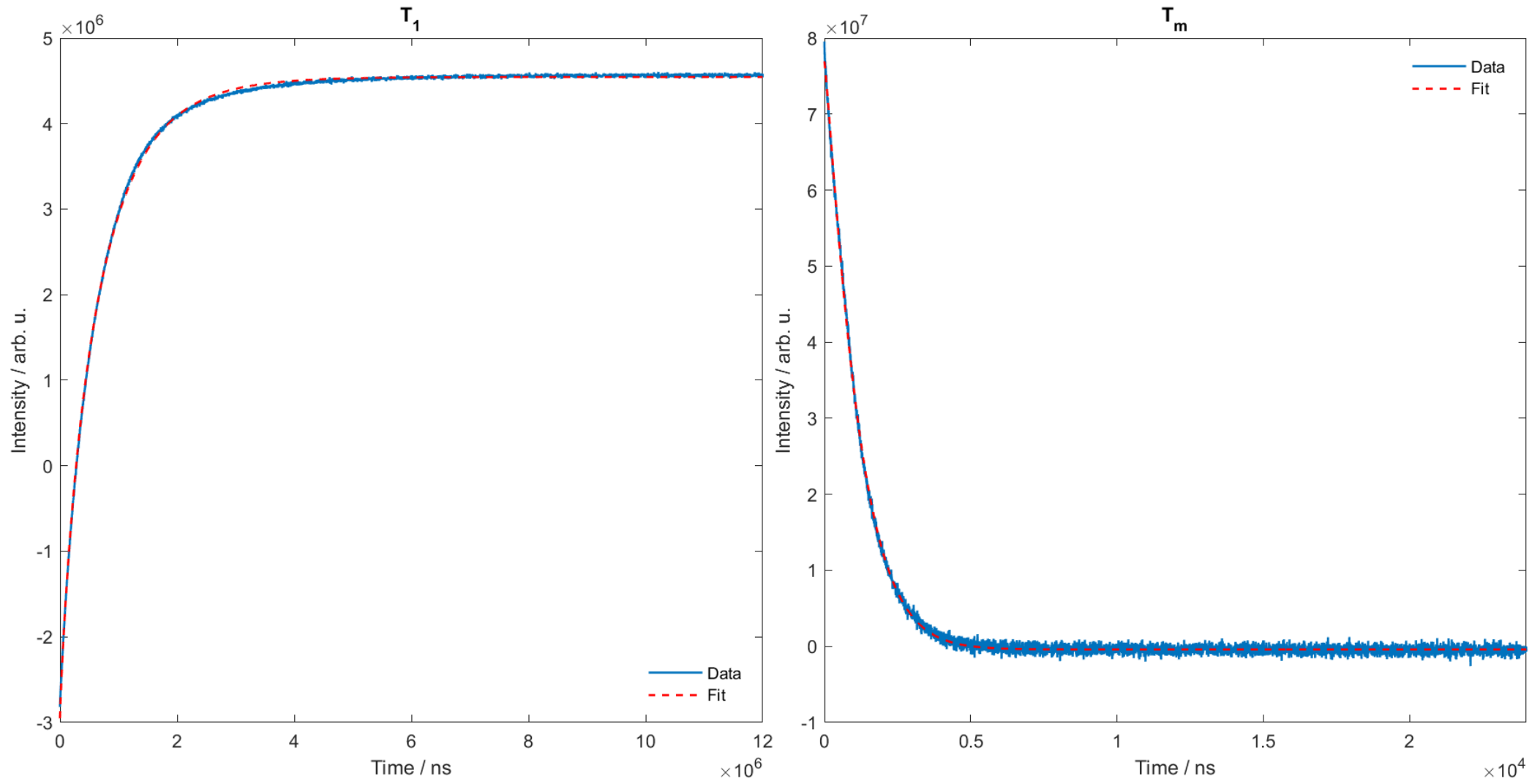

Figure S5. Spin lattice (T_1; left) and phase memory (T_m; right) relaxation time data for a 1 mM $^nPr_2O$ frozen solution of **2La** measured at B_0 = 342.0 mT and at 20 K.

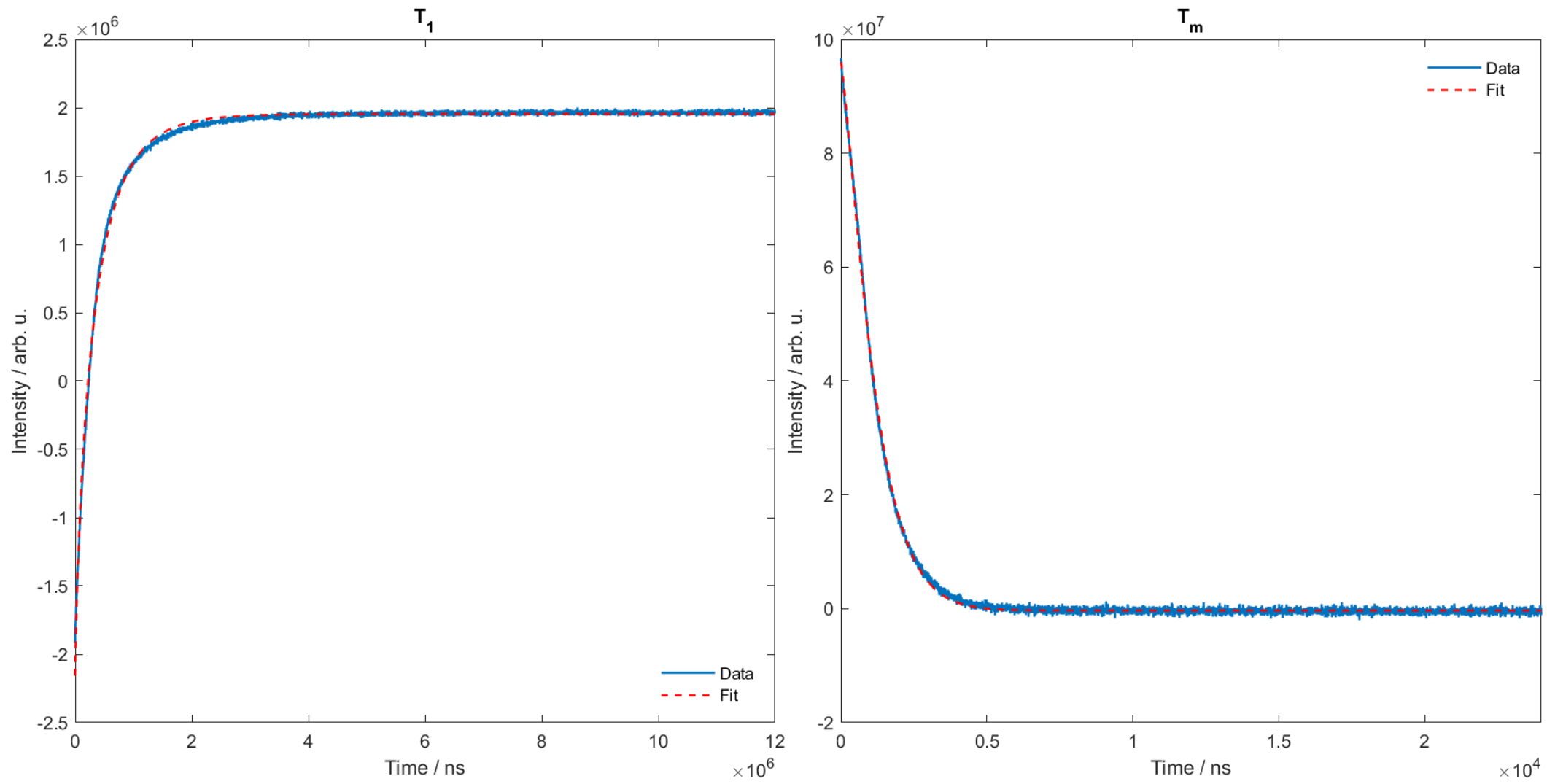

Figure S6. Spin lattice relaxation time (T_1; left) and phase memory (T_m; right) relaxation time data for a 1 mM $^nPr_2O$ frozen solution of **2La** measured at B_0 = 349.3 mT and at 20 K.

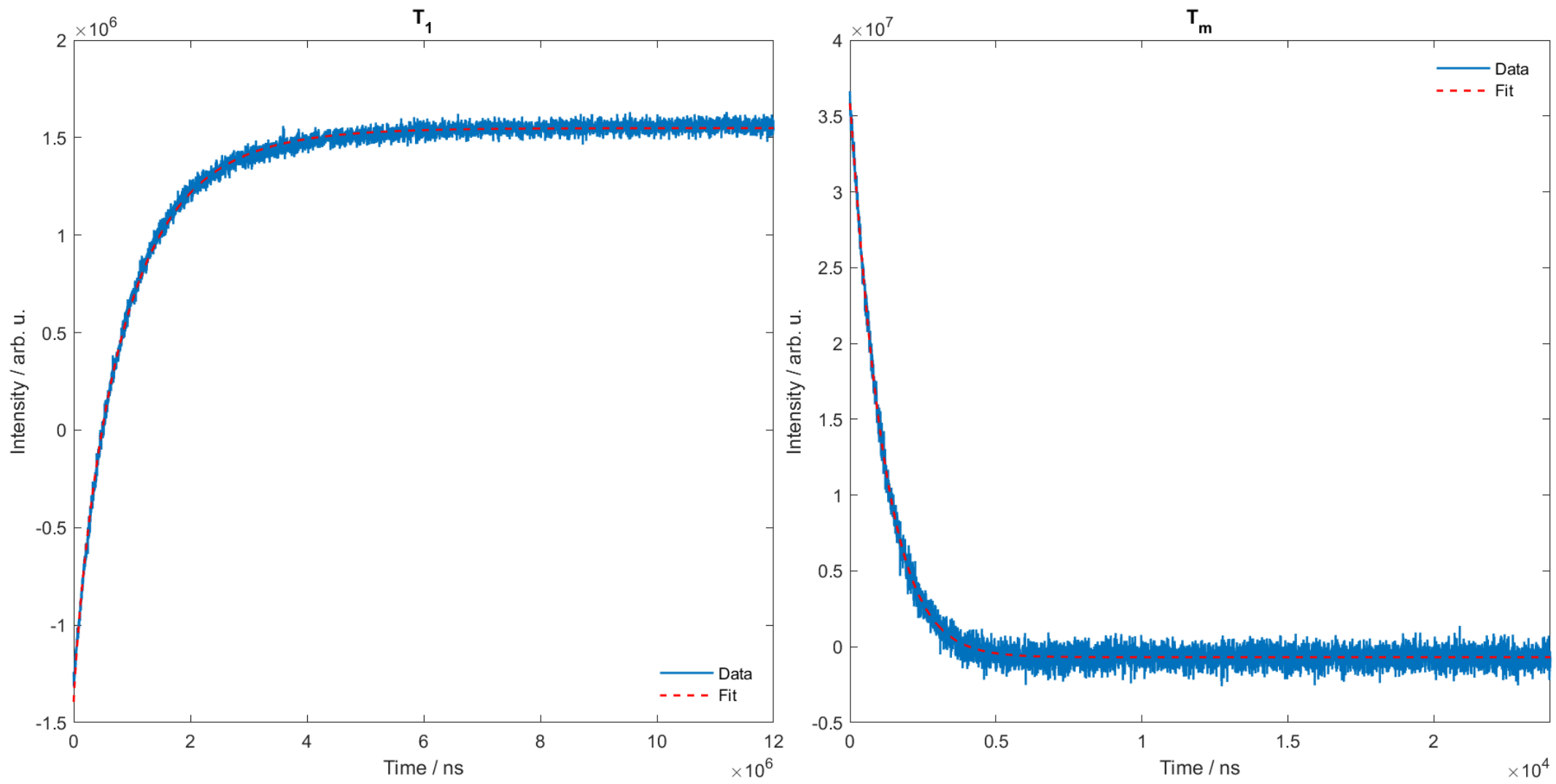

Figure S7. Spin lattice (T_1; left) and phase memory (T_m; right) relaxation time data for a 1 mM $^nPr_2O$ frozen solution of **2La** measured at B_0 = 368.0 mT and at 20 K.

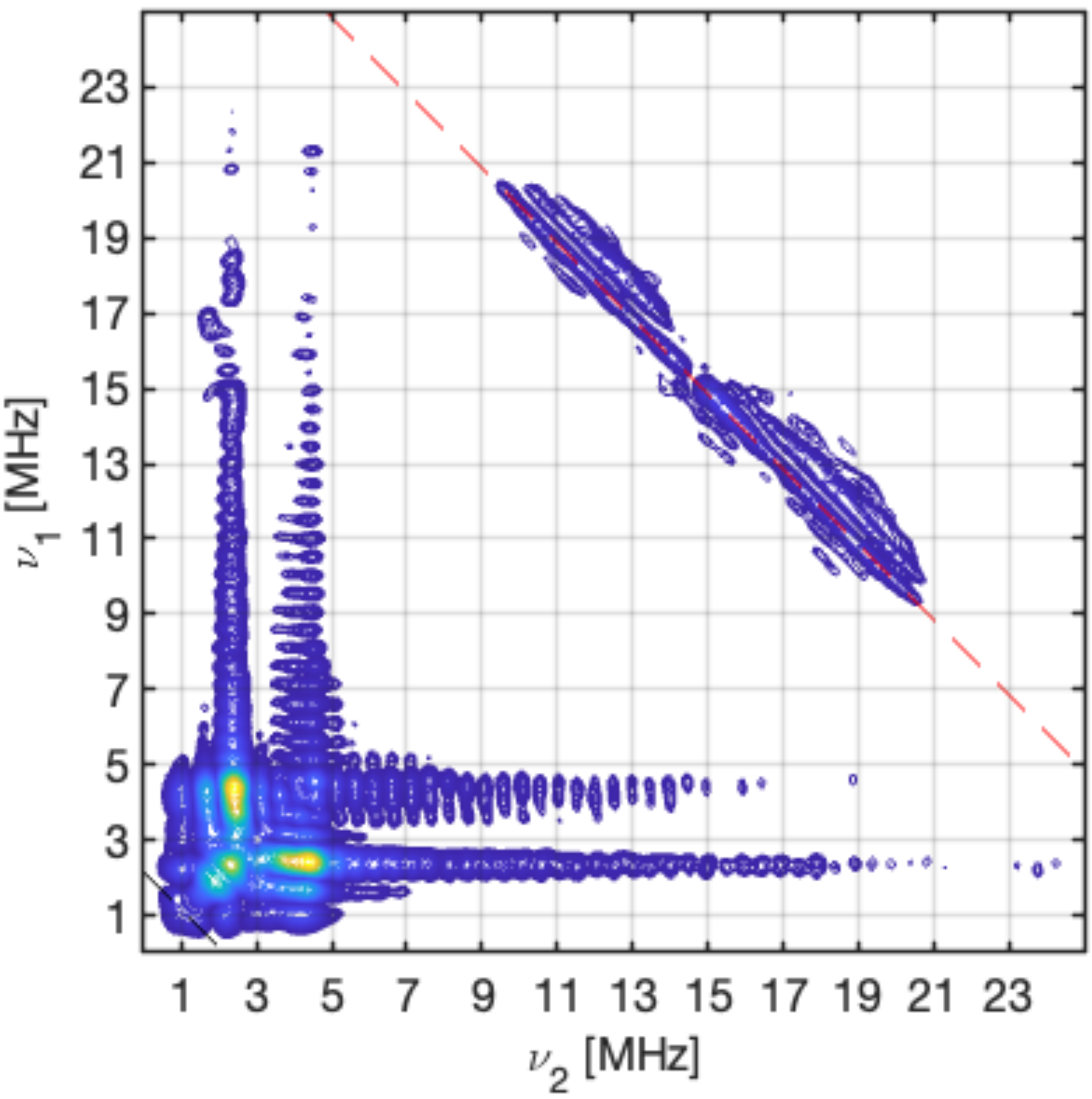

Figure S8. HYSCORE spectrum, showing ^1H and ^{14}N regions, of **2Sc** in 1 mM $^nPr_2O$ frozen solution measured at the maximum of the EDFS spectrum and at 40 K.

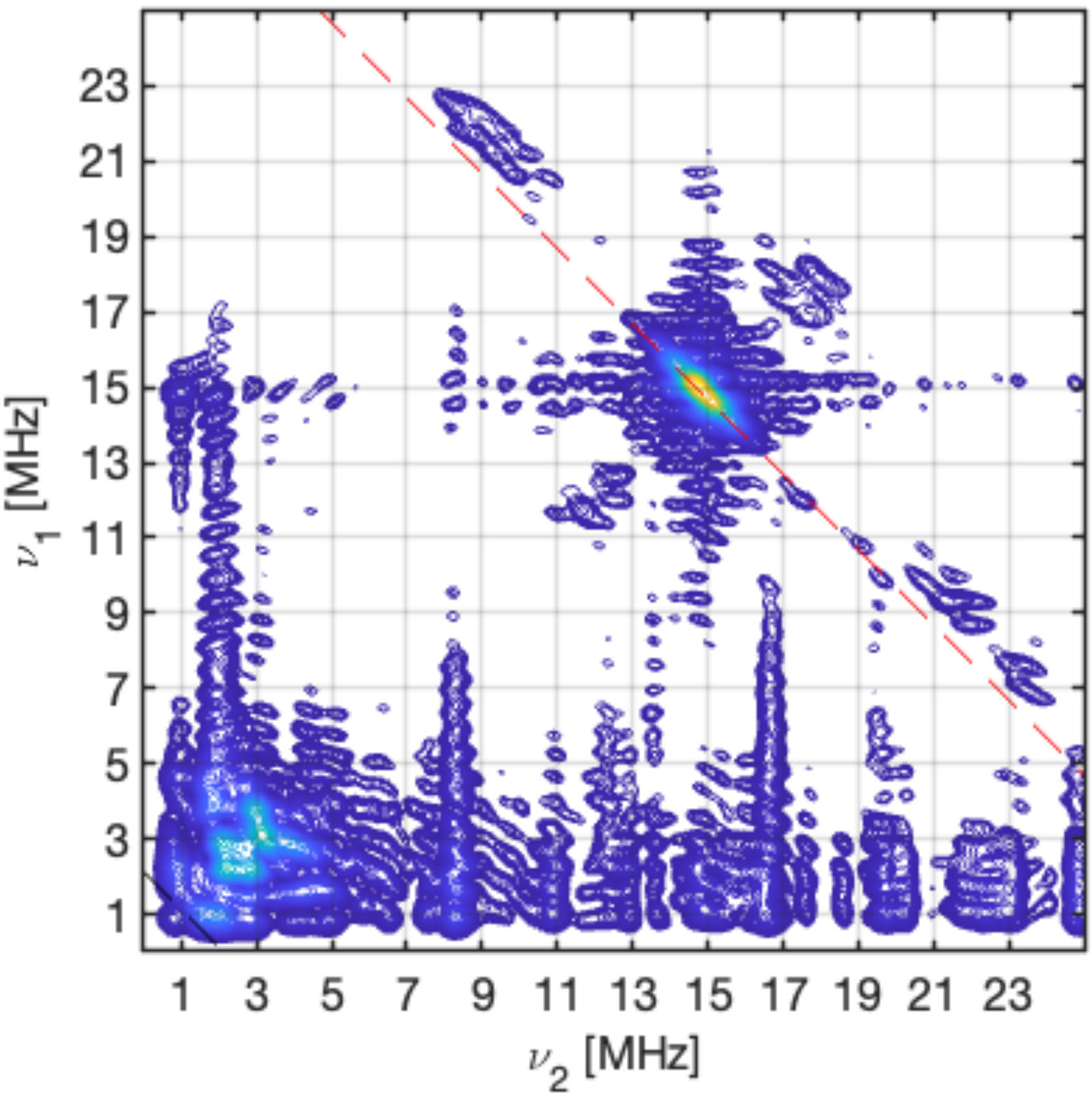

Figure S9. HYSCORE spectrum, showing ^{1}H and ^{14}N regions, of **2Y** in 1 mM $^{n}Pr_2O$ frozen solution measured at the maximum of the EDFS spectrum and at 20 K.

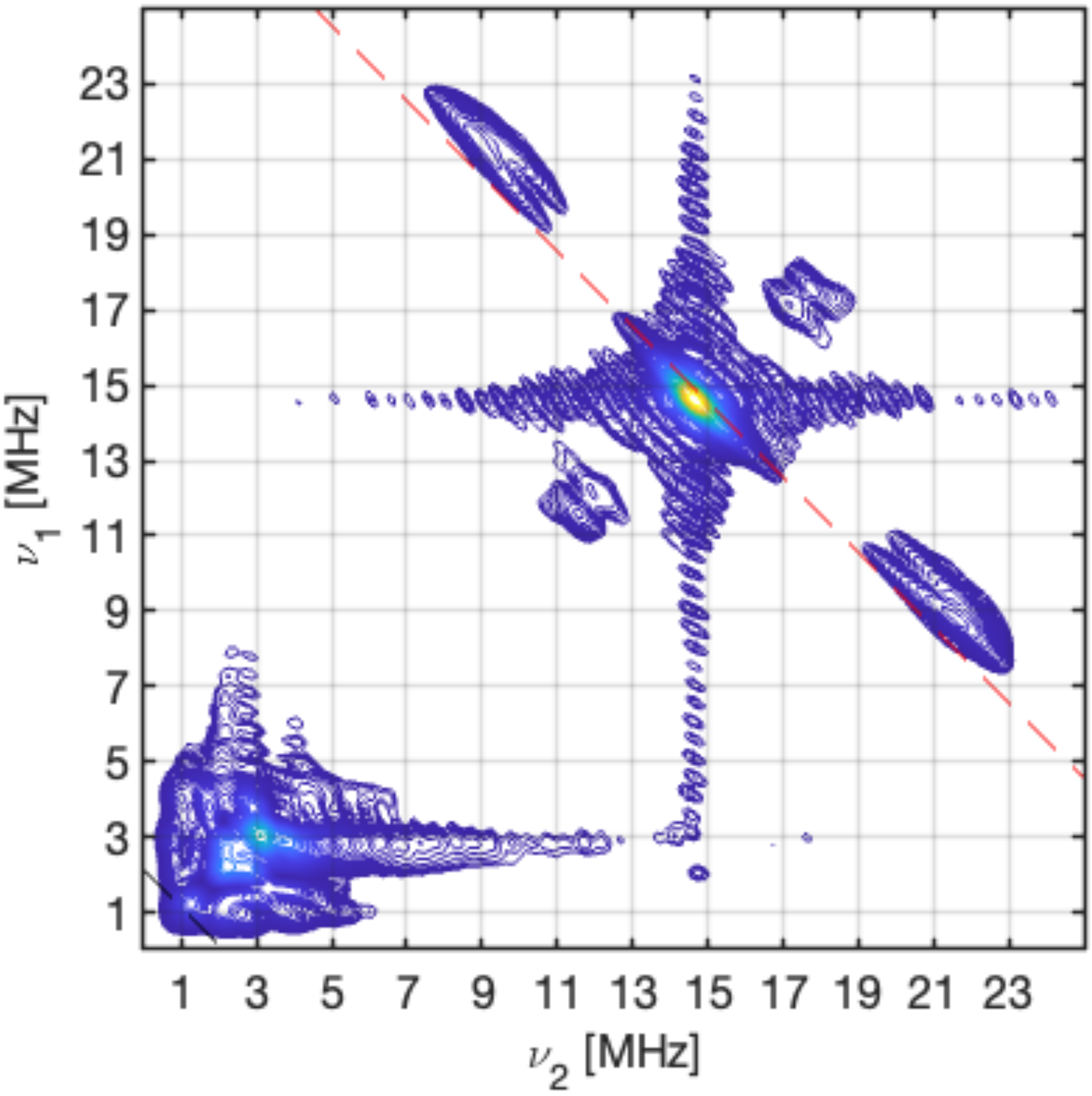

Figure S10. HYSCORE spectrum, showing ^{1}H and ^{14}N regions, of **2La** in 1 mM $^{n}Pr_2O$ frozen solution measured at the maximum of the EDFS spectrum and at 20 K.

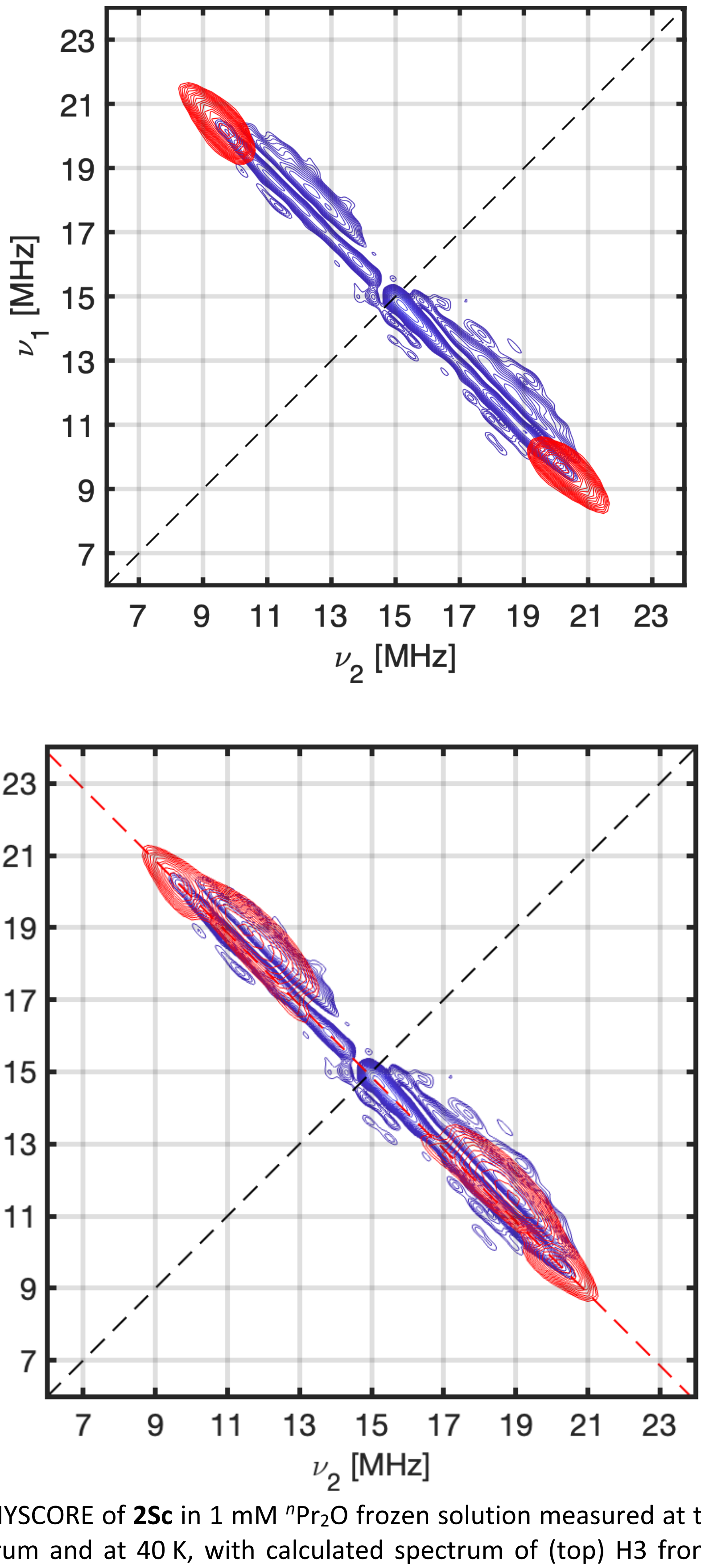

Figure S11. ^{1}H HYSCORE of **2Sc** in 1 mM $^{n}Pr_2O$ frozen solution measured at the maximum of the EDFS spectrum and at 40 K, with calculated spectrum of (top) H3 from Table S3, and (bottom) including all of H1-H4. Note that although H3 has a large $|A_3|$ component, because $|A_{iso}|$ is large, it only produces intensity near the extremity of the ridges.

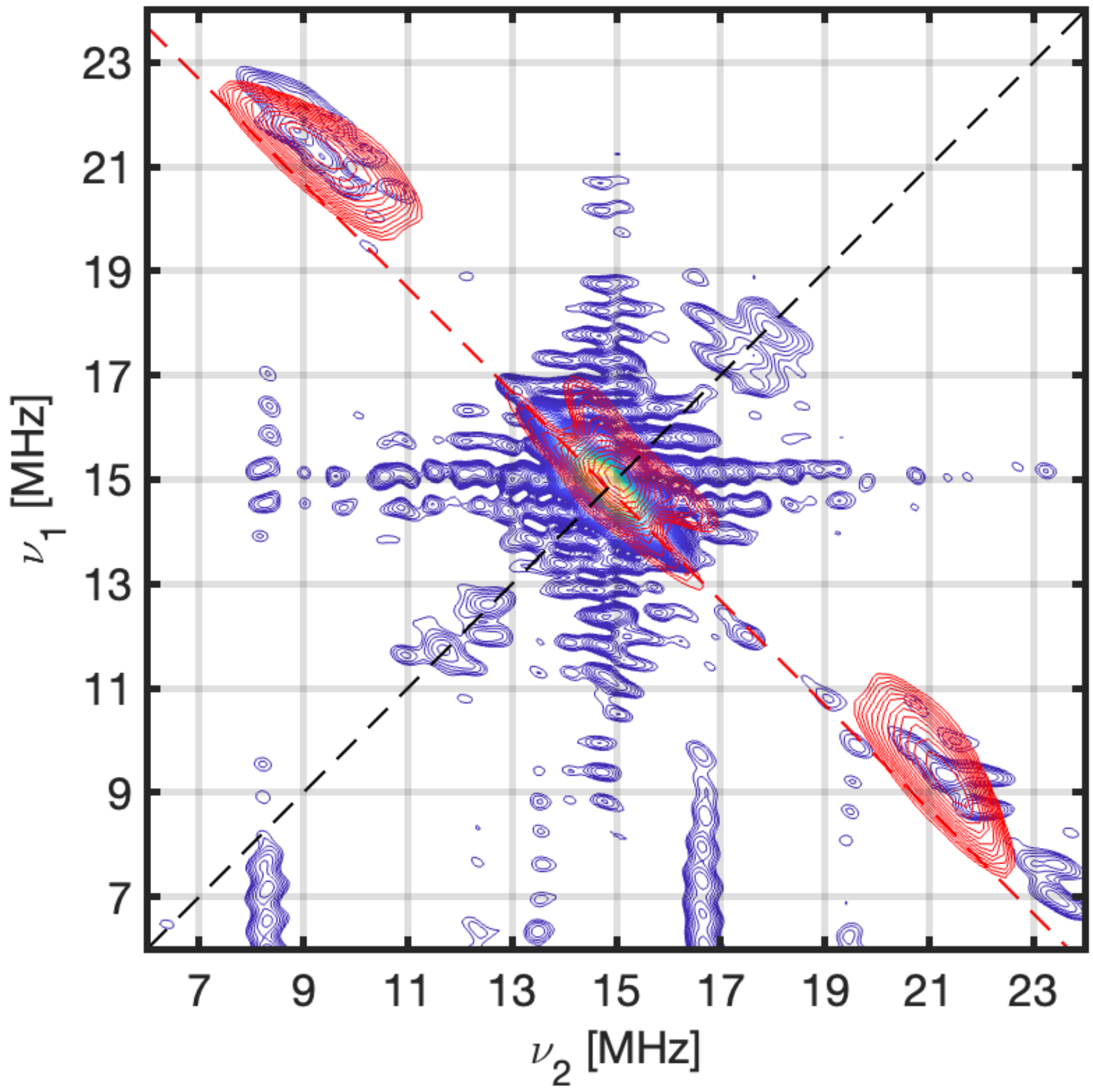

Figure S12. ^{1}H HYSCORE of **2Y** in 1 mM $^{n}Pr_2O$ frozen solution measured at the maximum of the EDFS spectrum and at 20 K, and calculated spectrum including H1-H4 from Table S4.

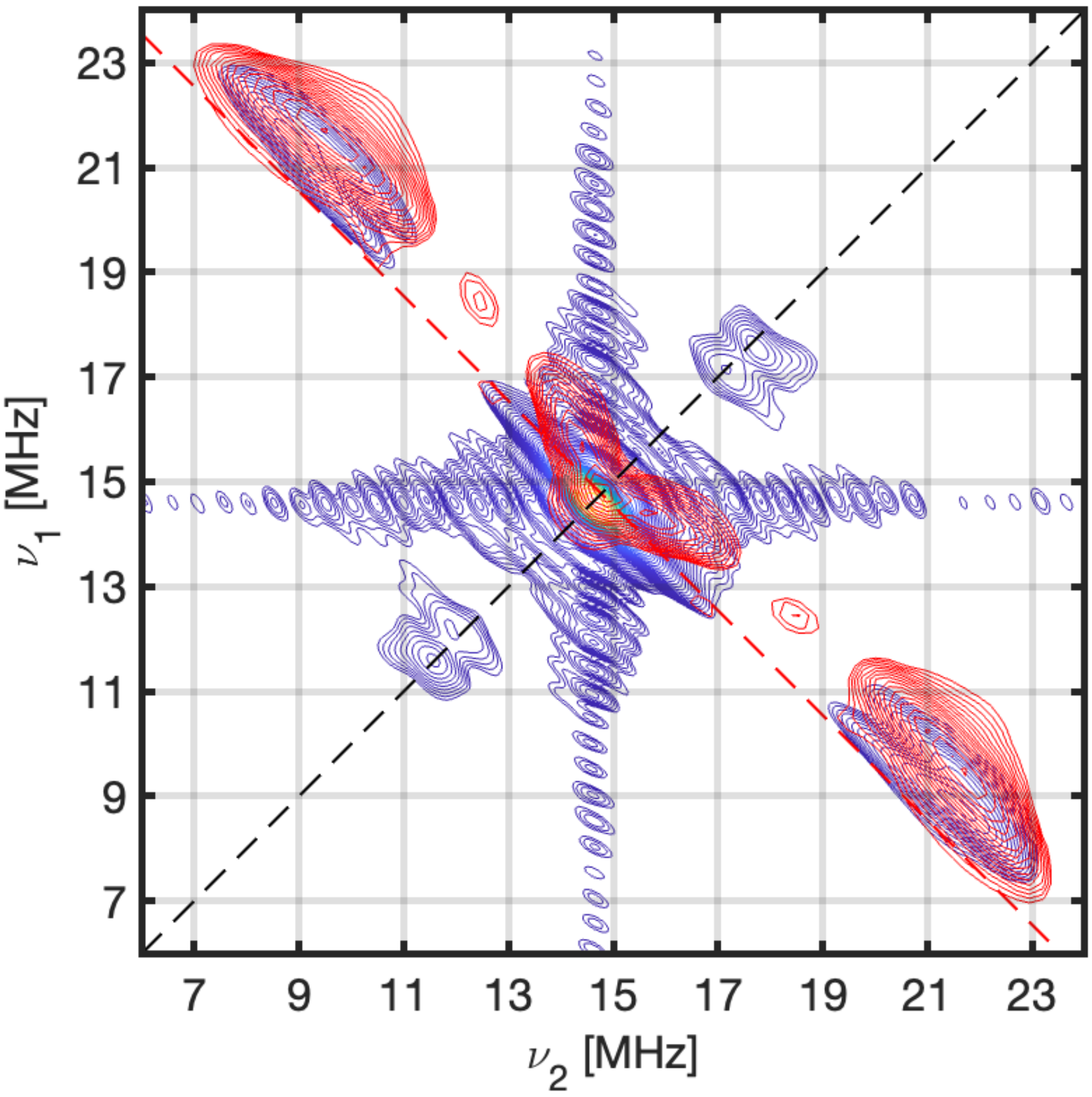

Figure S13. ^{1}H HYSCORE of **2La** in 1 mM ^{n}Pr$_2$O frozen solution measured at the maximum of the EDFS spectrum and at 20 K, and calculated spectrum including H1-H4 from Table S5.

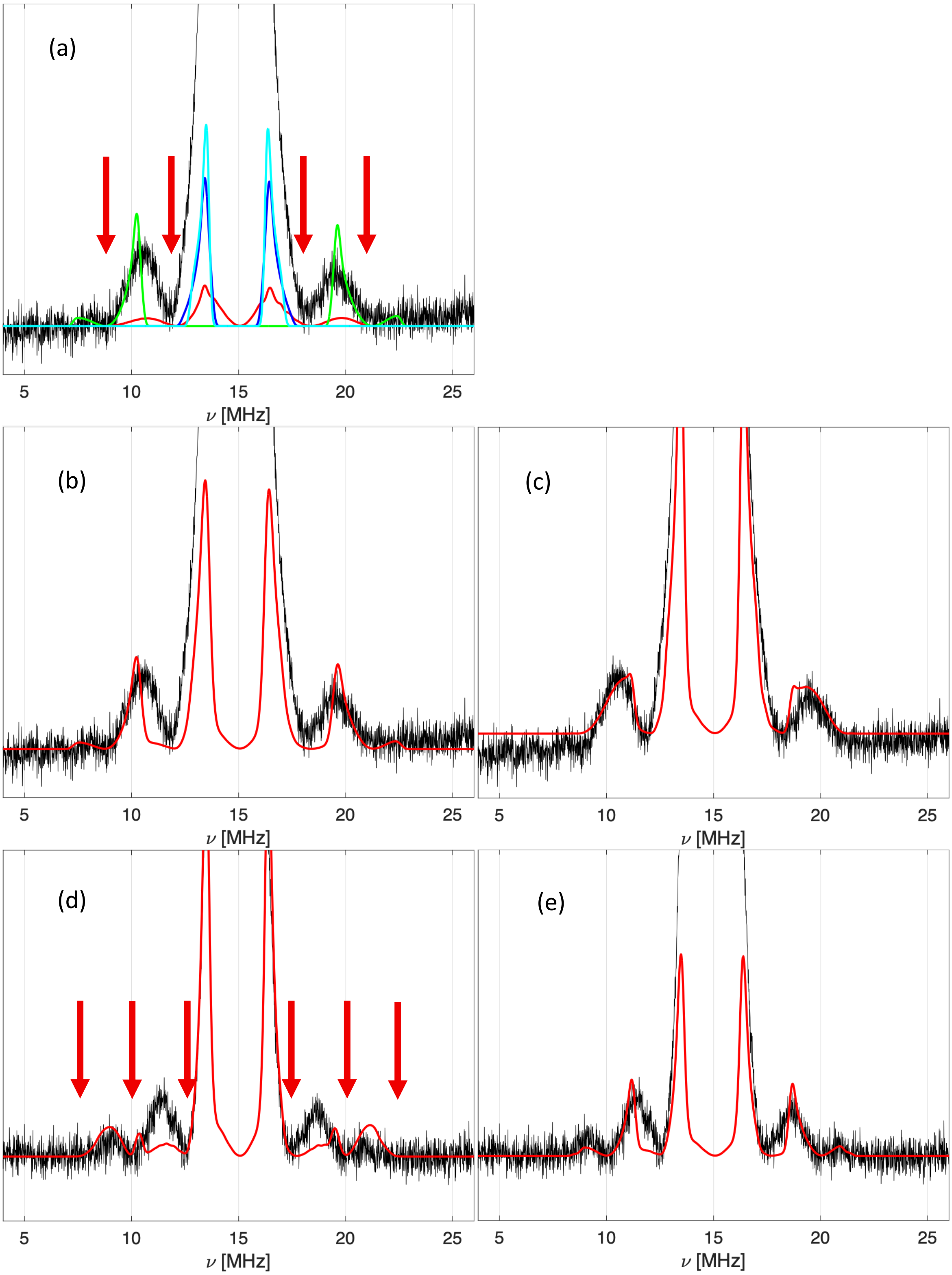

Figure S14. (a) Mims ENDOR spectrum of **2Sc** in 1 mM $^{n}Pr_2O$ solution at 40 K, measured at maximum of the EDFS (black; the intense central peaks due to numerous weak hyperfine couplings, are cropped). Calculated spectra for H2 (red), H3 (green), H4 (blue) and H1 (cyan), (Tables S2,S3). Arrows indicate blindspots for τ = 160 ns. (b) Summation of calculated spectra for H1-H4 (red) for τ = 160 ns. (c) As for (b), but with hyperfine matrix for H3 scaled by 0.8. (d,e) As for (b,c) but for τ = 200 ns.

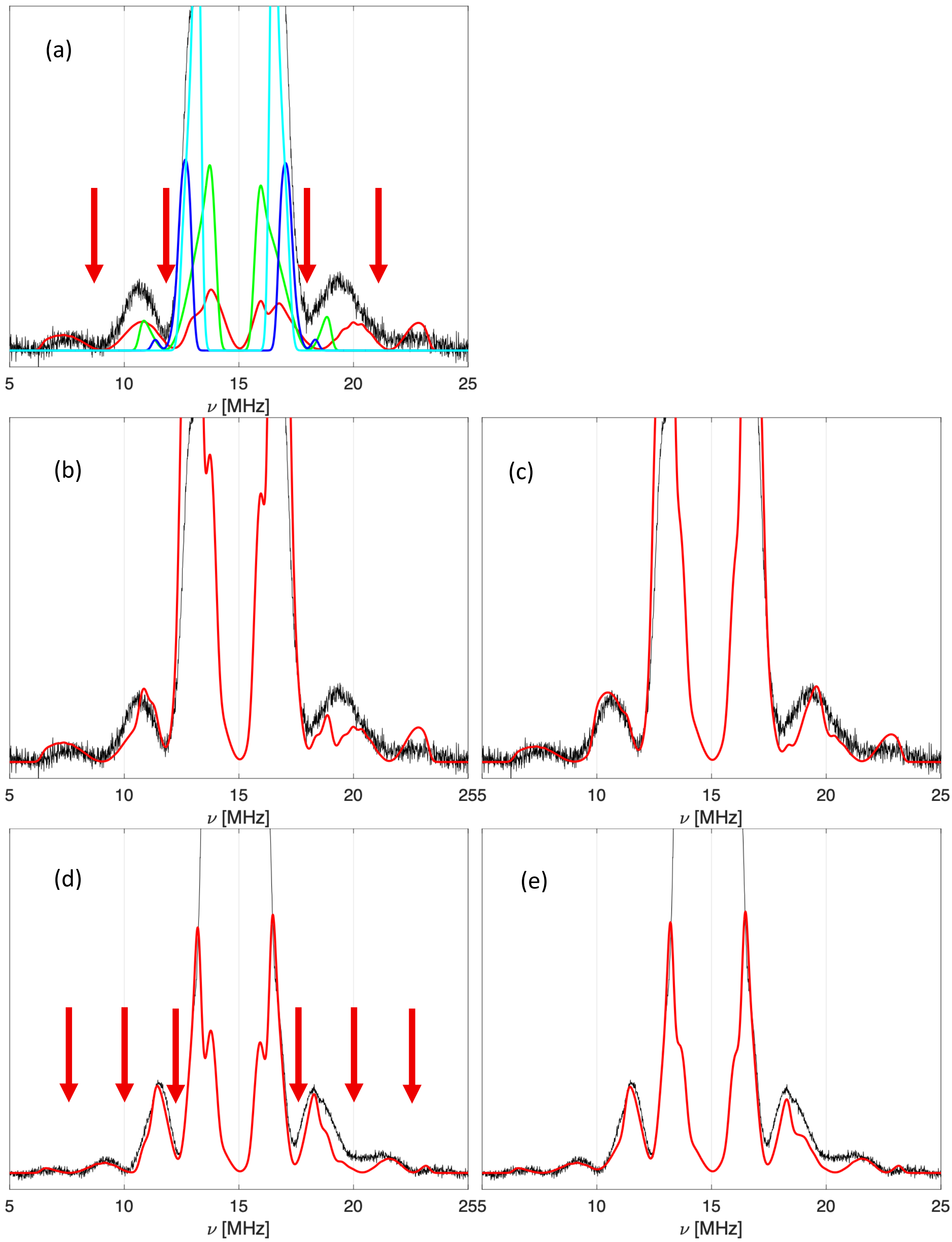

Figure S15. (a) Mims ENDOR spectrum of **2Y** in 1 mM $^{n}Pr_2O$ solution at 20 K, measured at maximum of the EDFS (black; the intense central peaks due to numerous weak hyperfine couplings are cropped). Calculated spectra for H2 (red), H3 (green), H4 (blue) and H1 (cyan) (Tables S2,S4). Arrows indicate blindspots for τ = 160 ns. (b) Summation of calculated spectra for H1-H4 (red) for τ = 160 ns. (c) As for (b), but with hyperfine matrix for H3 scaled by 1.2. (d,e) As for (b,c) but for τ = 200 ns.

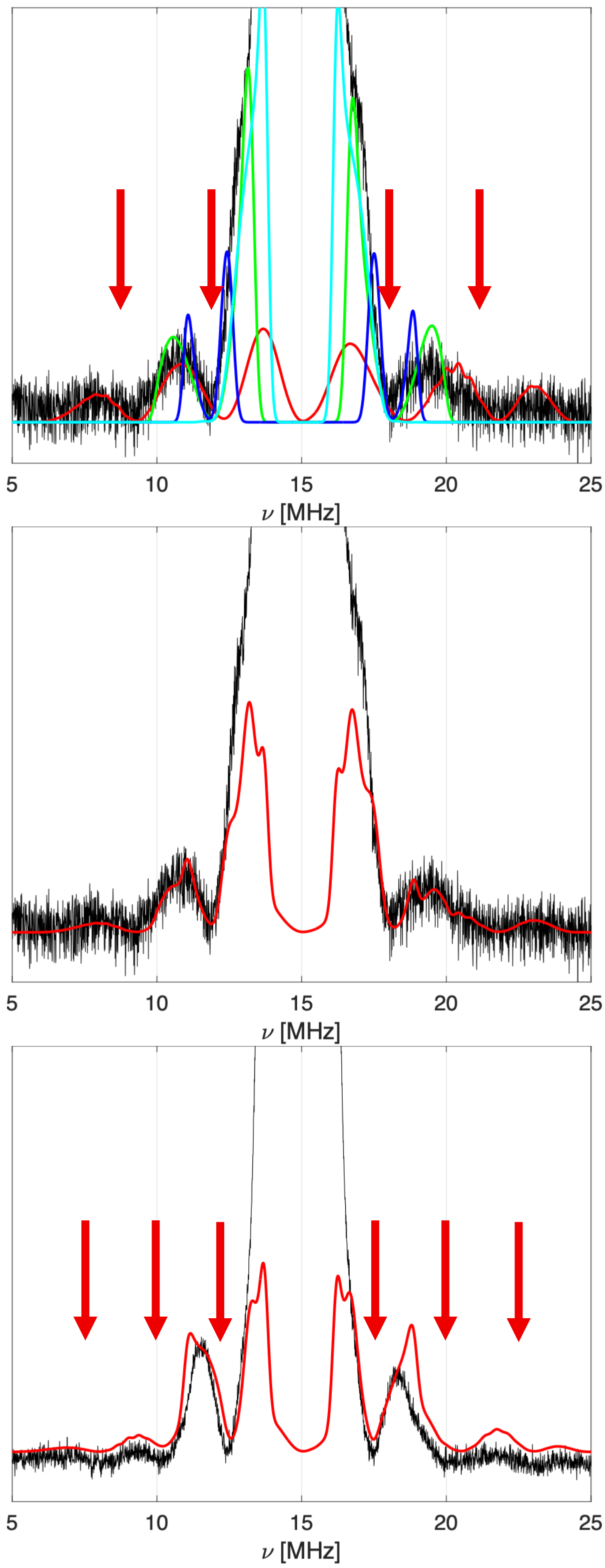

Figure S16. Top: Mims ENDOR spectrum of **2La** in 1 mM $^{n}Pr_2O$ solution at 20 K, measured at maximum of the EDFS spectrum (black; the intense central peaks due to numerous weak hyperfine couplings, are cropped). Calculated spectra for H2 (red), H3 (green), H4 (blue) and H1 (cyan) (Tables S2,S5). Arrows indicate blindspots for τ = 160 ns. Middle: Summation of calculated spectra for H1-H4 (red) for τ = 160 ns. Bottom: As for middle, but for τ = 200 ns, with blindspots shown.

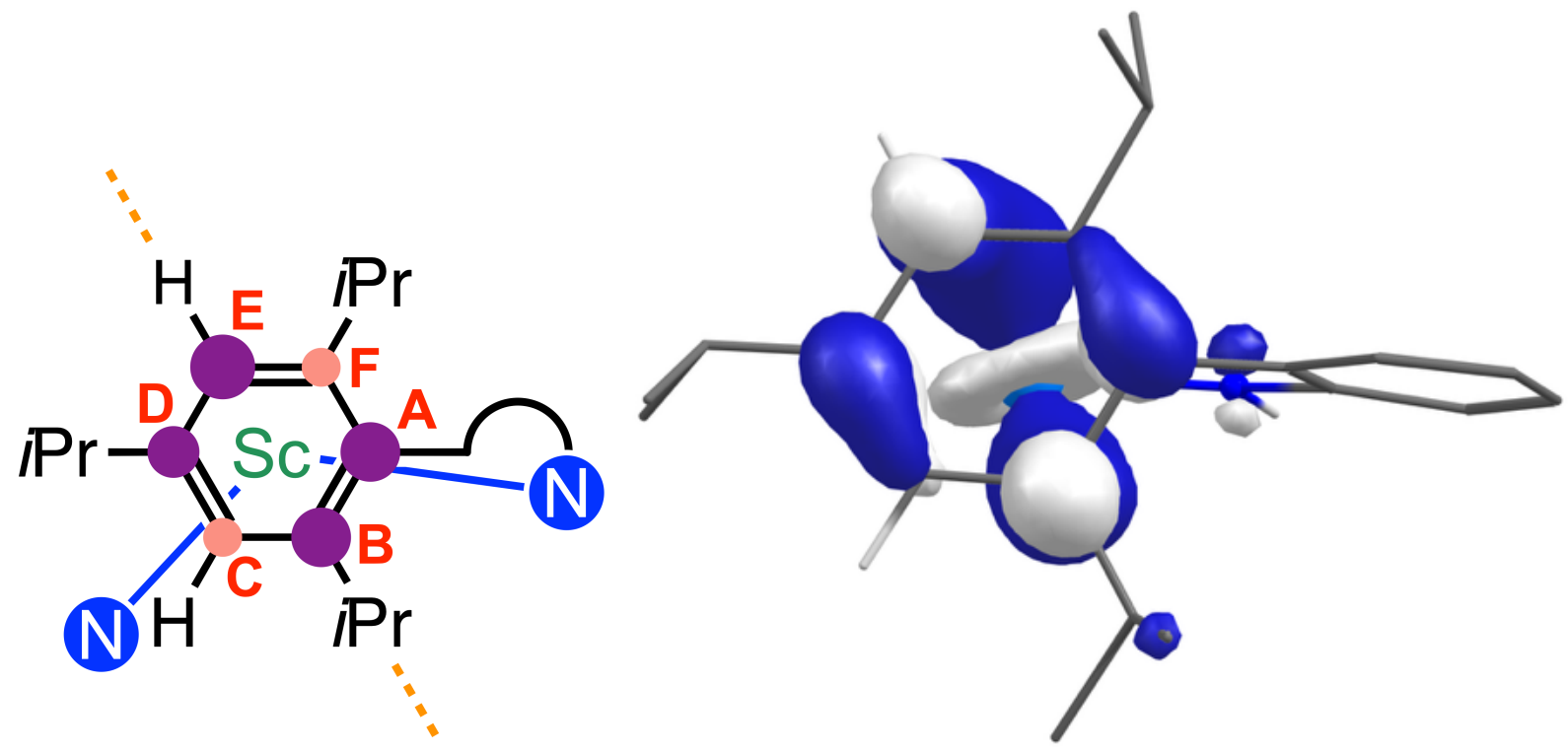

Figure S17. Schematic representation of the spin density distribution in **2Sc** closely bound Tripp group: purple – positive spin density; red – negative spin density; dashed orange line denotes Tripp hinge location (left). Visual representation of the calculated SOMO with an isovalue = 0.05, using the geometry derived from single crystal X-ray diffraction (right).

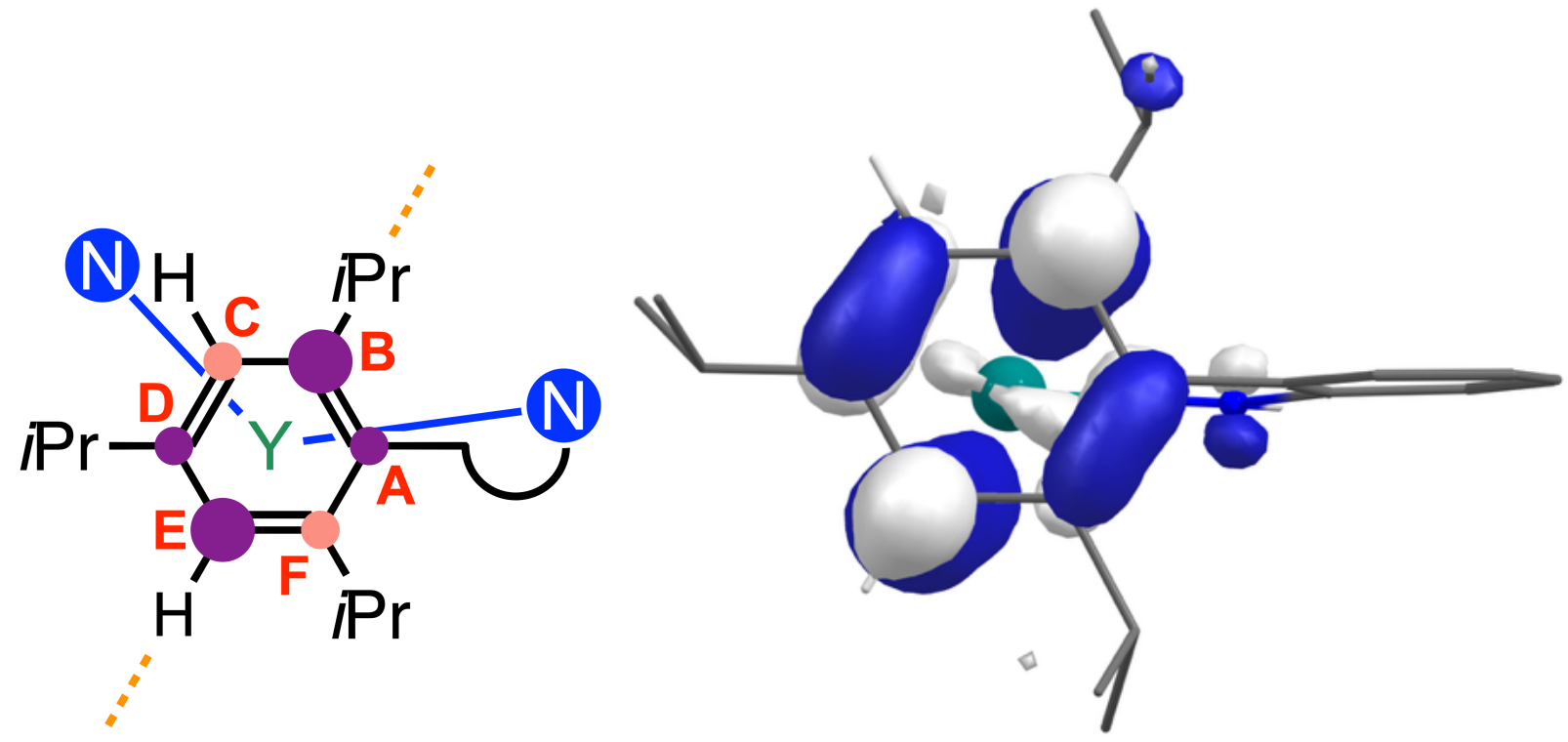

Figure S18. Schematic representation of the spin density distribution in **2Y** closely bound Tripp group: purple – positive spin density; red – negative spin density; dashed orange line denotes Tripp hinge location (left). Visual representation of the calculated SOMO with an isovalue = 0.05, using the geometry derived from the geometry-optimised structure of **2Y-Et$_2$O** (right).

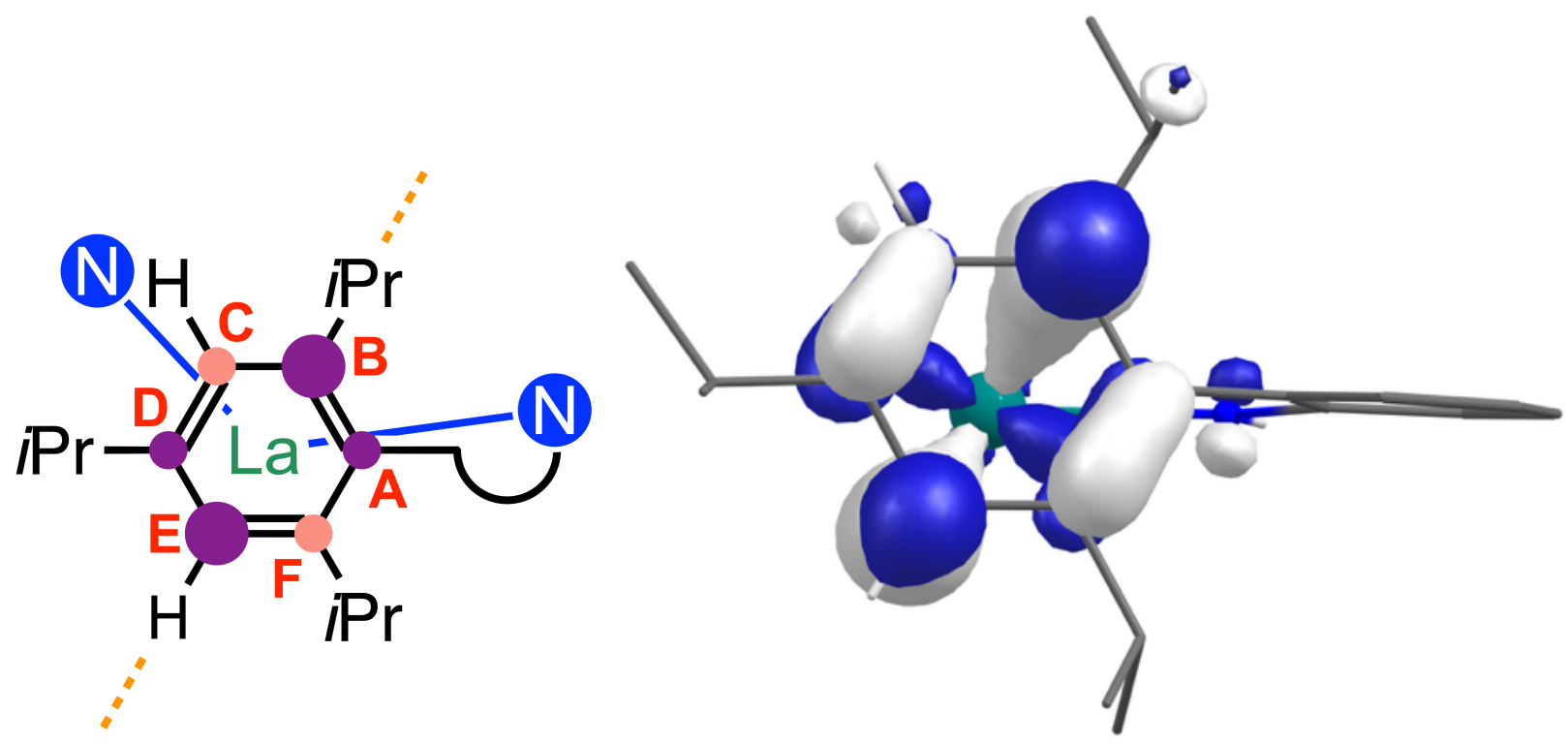

Figure S19. Schematic representation of the spin density distribution in **2La** closely bound Tripp group: purple – positive spin density; red – negative spin density; dashed orange line denotes Tripp hinge location (left). Visual representation of the calculated SOMO with an isovalue = 0.05, using the geometry derived from single crystal X-ray diffraction (right).

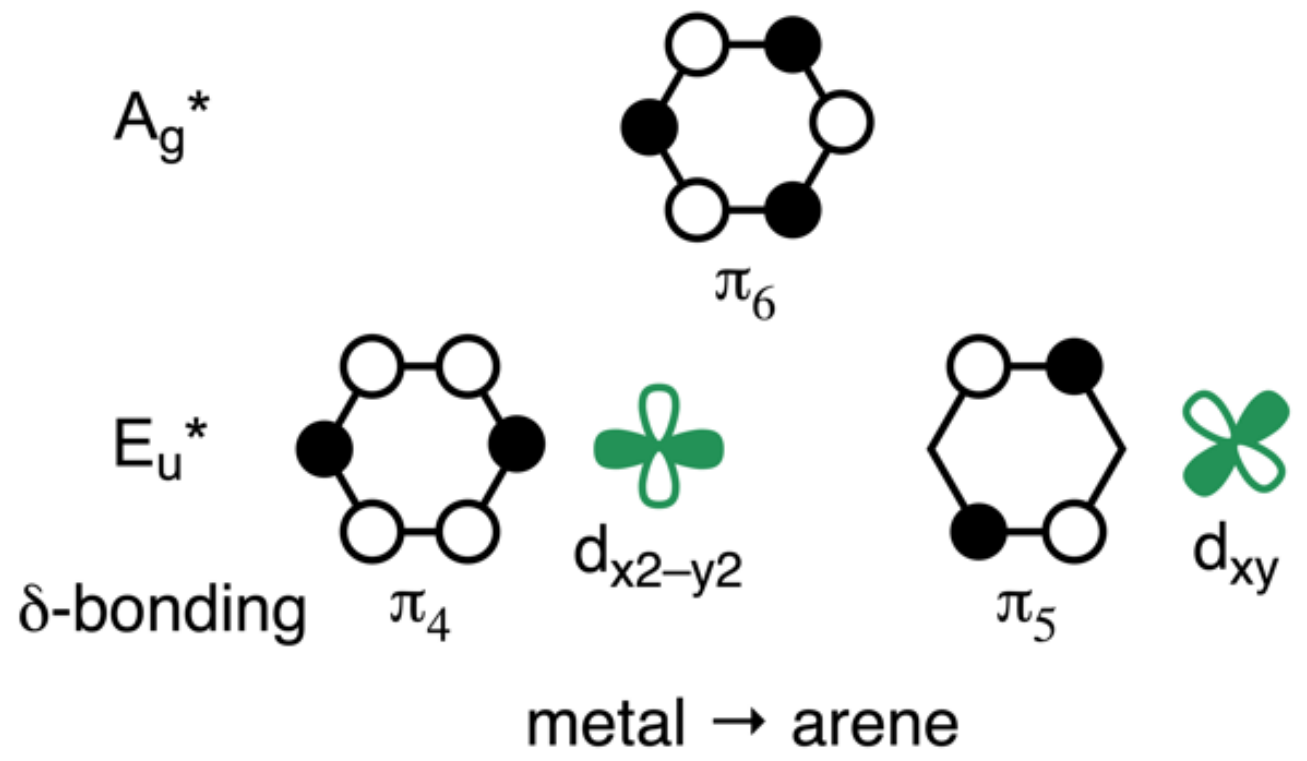

Figure S20. An illustration of metal-arene symmetry allowed orbital interactions for a d^0 metal, showing the metal → arene donation.

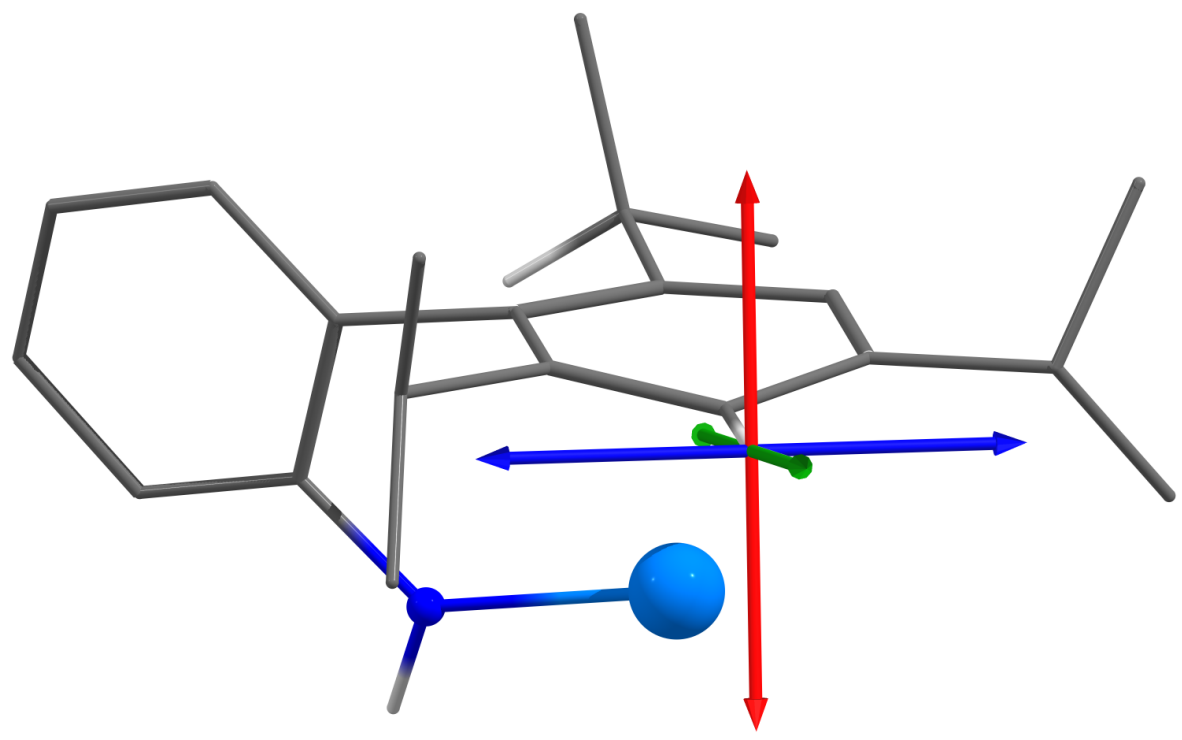

Figure S21. Principal *A*-tensor orientations for the *meta* H-atom (H2, Fig. 4) in **2Sc**. A_1 (green), A_2 (red) and A_3 (blue; see Table 2) projections are indicated by the arrows. The molecule has been truncated for visibility.

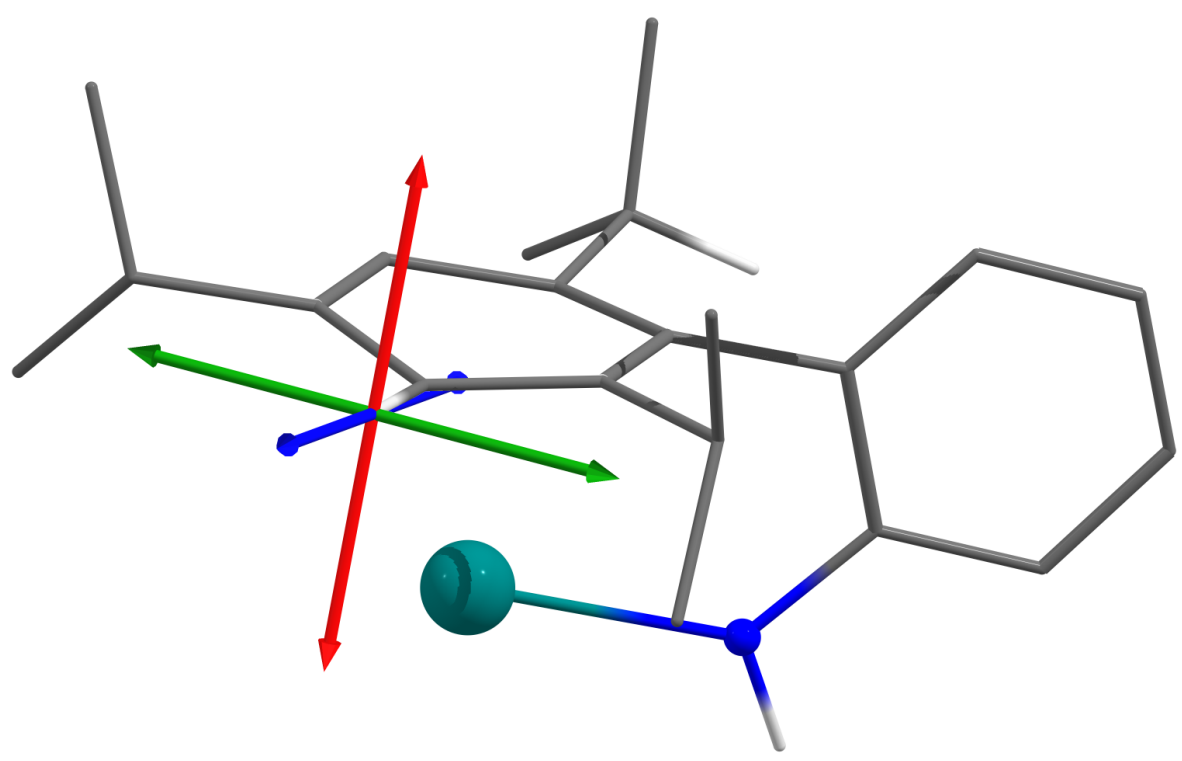

Figure S22. Principal *A*-tensor orientations for the *meta* H-atom (H2, Fig. 4) in **2Y**. A_1 (blue), A_2 (red) and A_3 (green; see Table 2) projections are indicated by the arrows. The molecule has been truncated for visibility.

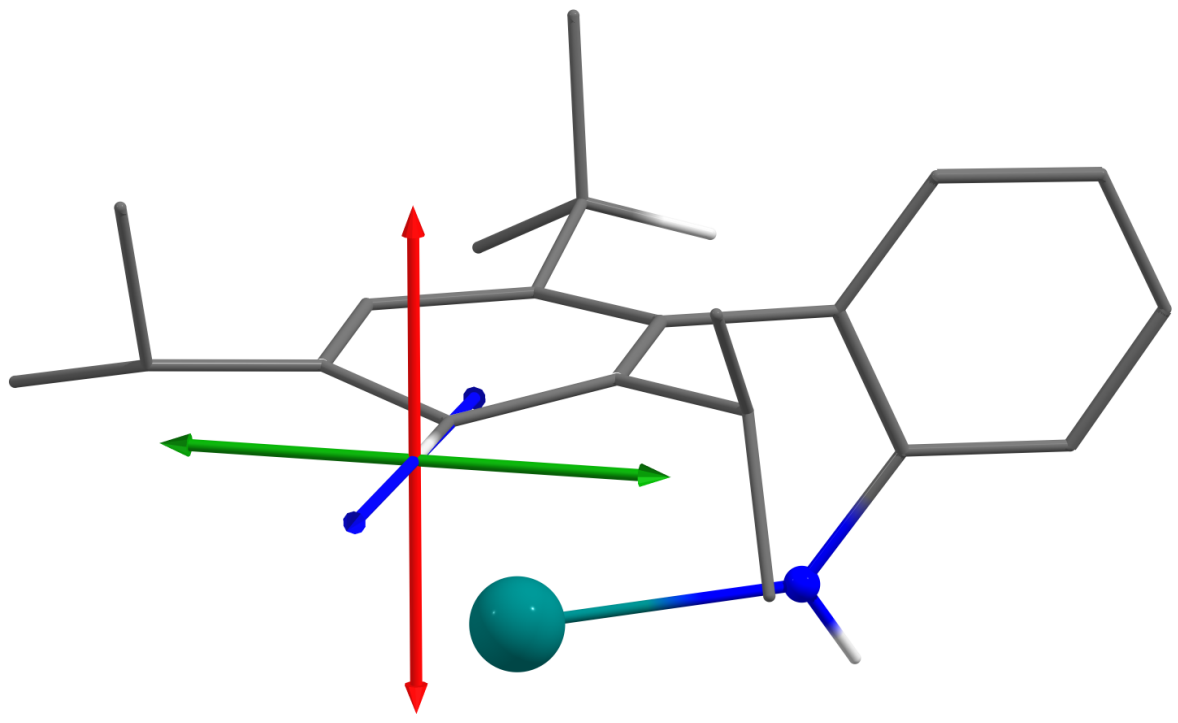

Figure S23. Principal *A*-tensor orientations for the *meta* H-atom (H2, Fig. 4) in **2La**. A_1 (blue), A_2 (red) and A_3 (green; see Table 2) projections are indicated by the arrows. The molecule has been truncated for visibility

References

1. R. E. MacKenzie, T. Hajdu, J. A. Seed, G. F. S. Whitehead, R. W. Adams, N. F. Chilton, D. Collison, E. J. L. McInnes and C. A. P. Goodwin, *Chem. Sci.* 2024, **15**, 15160-15169.
2. S. Van Doorslaer, *eMagRes*, 2017, **6**, 51–70.
3. J. R. Harmer, *eMagRes*, 2016, **5**, 1493–1514.
4. S. Stoll and A. Schweiger, *J. Magn. Reson.* 2006, **178**, 42-55; S. Stoll and R. D. Britt, *Phys. Chem. Chem. Phys.* 2009, **11**, 6614-6625.
5. V. N. Staroverov, G. E. Scuseria, J. Tao and J. P. Perdew, *J. Chem. Phys.* 2003, **119**, 12129-12137; *ibid.* 2004, **121**, 11507.
6. J. Tao, J. P. Perdew, V. N. Staroverov and G. E. Scuseria, *Phys. Rev. Lett.*, 2003, **91**, 146401.
7. S. Grimme, J. Antony, S. Ehrlich and H. Krieg, *J. Chem. Phys.*, 2010, **132**, 154104-154104–19.
8. S. Grimme, *J. Comput. Chem.*, 2006, **27**, 1787–1799.
9. S. Grimme, S. Ehrlich and L. Goerigk, *J. Comput. Chem.*, 2011, **32**, 1456–1465.
10. F. Neese, F. Wennmohs, A. Hansen and U. Becker, *Chem. Phys.*, 2009, **356**, 98–109.
11. R. Izsak and F. Neese, *J. Chem. Phys.*, 2011, **135**, 144105.
12. D. Aravena, F. Neese and D. A. Pantazis, *J. Chem. Theory Comput*. 2016, **12**, 1148-1156.
13. D. A. Pantazis and F. Neese, *J. Chem. Theory Comput*. 2009, **5**, 2229-2238.
14. D. A. Pantazis, X. Y. Chen, C. R. Landis and F. Neese, *J. Chem. Theory Comput*. 2008, **4**, 908-909.
15. F. Wiegend, *Phys. Chem. Chem. Phys.* 2006, **8**, 1057-1065.
16. D. A. Pantazis and F. Neese, *J. Chem. Theory Comput*. 2011, **7**, 677-684.
